# Microfluidic‐Assisted Evolution of a Robust NAD^+^‐Dependent Enzyme with Improved Isobutanol Tolerance at Elevated Temperatures

**DOI:** 10.1002/cssc.202501120

**Published:** 2025-07-10

**Authors:** Mariko Teshima, Robert Genth, Tenuun Bayaraa, Manuel Döring, Barbara Beer, Gerhard Schenk, Volker Sieber

**Affiliations:** ^1^ Chair of Chemistry of Biogenic Resources Technical University of Munich Campus Straubing for Biotechnology and Sustainability Schulgasse 16 94315 Straubing Germany; ^2^ School of Chemistry and Molecular Biosciences The University of Queensland 68 Cooper Rd St. Lucia Brisbane 4072 Australia; ^3^ Australian Institute for Bioengineering and Nanotechnology The University of Queensland Corner of College and Cooper Rds St. Lucia Brisbane 4072 Australia; ^4^ Sustainable Minerals Institute The University of Queensland Corner of College and Staff House Rds St. Lucia Brisbane 4072 Australia; ^5^ SynBioFoundry@TUM Technical University of Munich Schulgasse 22 94315 Straubing Germany; ^6^ Catalytic Research Center Technical University of Munich Ernst‐Otto‐Fischer Straße 1 85748 Garching Germany; ^7^ Present address: Fluidic Disposal Development (DSRPDD) Roche Diagnostics GmbH Sandhofer Str. 116 68305 Mannheim Germany; ^8^ Present address: CASCAT GmbH Europaring 4 94315 Straubing Germany

**Keywords:** absorbance‐activated droplet sorting, aldehyde dehydrogenase, directed evolution, isobutanol, thermostability

## Abstract

Prompted by the thermostability issue identified in recent work on enzyme discovery/engineering and its application, the directed evolution of an NAD^+^‐dependent aldehyde dehydrogenase (ALDH) with improved thermostability and isobutanol tolerance at 50 °C, properties required for its successful implementation in cell‐free isobutanol biosynthesis, is described herein. ALDH not only plays an important role in multienzyme cascades for the production of platform chemicals but also represents a bottleneck due to its modest stability. Using a custom‐built absorbance‐activated droplet sorter, ultrahigh‐throughput microfluidic screening of a randomized library of 63,000 members is performed, leading to the discovery of a variant with a 250‐fold prolonged half‐life at 50 °C without significant loss of activity. Subsequently, the most promising mutations are distributed on designer templates in the combinatorial staggered extension process library to create a new generation of variants. One of these variants shows a threefold increase in *k*_cat_ *K*_m_^−1^. Another shows significantly higher stability in 3% v/v isobutanol, retaining ≈50% of its initial activity after 6 h of incubation at 50 °C. Finally, a cell‐free multienzymatic cascade using the ultimate variant demonstrates its superior stability in 4% v/v isobutanol at 50 °C, highlighting the success of engineering to overcome the cofactor‐related challenge of establishing cascade balance.

## Graphical abstract

Utilizing the power of the absorbance‐activated droplet sorter, a key enzyme for a cell‐free multienzymatic cascade for sustainable isobutanol biosynthesis is optimized for improved thermostability and isobutanol tolerance required for efficient cascade performance. By combining extensive randomization using microfluidic screening and recombination of candidate mutations using designer templates, the enzyme half‐life is rapidly increased from 3 min to 12.7 h.

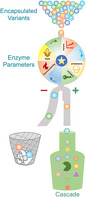

## 1 Introduction

Directed evolution is one of the most popular methods used for enzyme engineering, highlighted by the 2018 Nobel Prize in Chemistry.^[^ ^1^ ^]^ However, the success of this approach depends on exploring the maximum combinatorial diversity, which requires the screening of large mutant libraries.^[1a]^ This, in turn, requires highly efficient methods that screen several tens of thousands of variants per hour without accumulating large amounts of waste (e.g., microtiter plates, liquids, and expensive compounds for assays). To overcome the technical limitations of exploring a vast sequence space, a major trend in recent years has focused on the generation of “smart libraries” (semirational approach),^[^ ^2^ ^]^ assisted by novel bioinformatics tools, mainly based on sequence and protein structure data from various available databases.^[^ ^3^ ^]^ On the other hand, the integration of machine learning approaches into directed evolution protocols has enabled the efficient design of enzyme variants with high fitness levels.^[^ ^4^ ^]^ However, methods to screen the massive sequence diversity with high screening capacity remain indispensable. First, recent advances in molecular biology have enabled in vivo mutagenesis for continuous enzyme evolution without DNA isolation, in vitro DNA manipulation, and transformation of cells.^[^ ^5^ ^]^ Furthermore, genetically encoded biosensors such as (allosteric) transcription factors or riboswitches can be used in combination with screening using a fluorescence‐activated cell sorter.^[^ ^2^ ^]^ Second, technical advances have led to ultrahigh‐throughput screening (UHTS) using absorbance‐activated droplet sorter (AADS), which allows highly efficient screening in nano‐ to femtoliter volumes.^[^ ^6^ ^]^ This technical advance extended conventional fluorescence readouts to absorbance readouts.^[1a]^ In this approach, individual members of enzyme libraries are expressed in a suitable host such as *Escherichia coli* and compartmentalized in water‐in‐oil emulsion droplets together with a lysis agent and assay components.^[^ ^7^ ^]^ The droplets are incubated to allow a catalytic reaction to occur, and the droplets are subsequently sorted by absorbance readouts based on enzyme turnover.

This work demonstrates the engineering of a cofactor‐dependent key enzyme of a cell‐free multienzyme cascade for the sustainable, nonfossil‐based production of platform chemicals.^[^ ^8^ ^]^ In this artificial shortcut cascade based on the nonphosphorylative Entner–Doudoroff pathway and the substrate promiscuity of the dehydratase, the NAD^+^‐dependent aldehyde dehydrogenase (ALDH), which converts d‐glyceraldehyde to d‐glycerate (glyceraldehyde dehydrogenase, GADH), plays a significant role in the cofactor balance of a closed‐loop cascade and enzyme viability. After the successful optimization of cell‐free ethanol production through GADH engineering,^[8c]^ enzyme stability remained a major challenge for its successful extension to the isobutanol cascade. The application‐inspired ALDH optimization in the present work exploits the utility of the AADS—a technology that still needs to demonstrate its universal applicability for wider adoption in enzyme engineering—to explore broad, gene‐wide sequence variability to optimize stability. We address the challenges of library design, compatibility of enzyme assay at elevated temperatures with the operation of the AADS, and evaluation and sample tracking during repeated cycles of screening to extract maximum information from the AADS screening. The characterization of the engineered ALDH variants highlights their superior robustness, including their improved longevity under desired cascade conditions and increased tolerance to isobutanol, a platform chemical of great interest to the biofuel and bioplastics industries.^[^ ^9^ ^]^ In addition, some variants also displayed improved catalytic efficiency. Finally, the cell‐free ethanol cascade in the presence of 4% v/v isobutanol confirmed the functionality and superior stability of the ultimate variant. This variant would open the possibility of extending the biocatalytic process toward our future goal of developing a robust green production of isobutanol.

## 2 Results and Discussion

### 2.1 Microfluidic Screening of an Error‐Prone PCR Library

**Figure 1:**
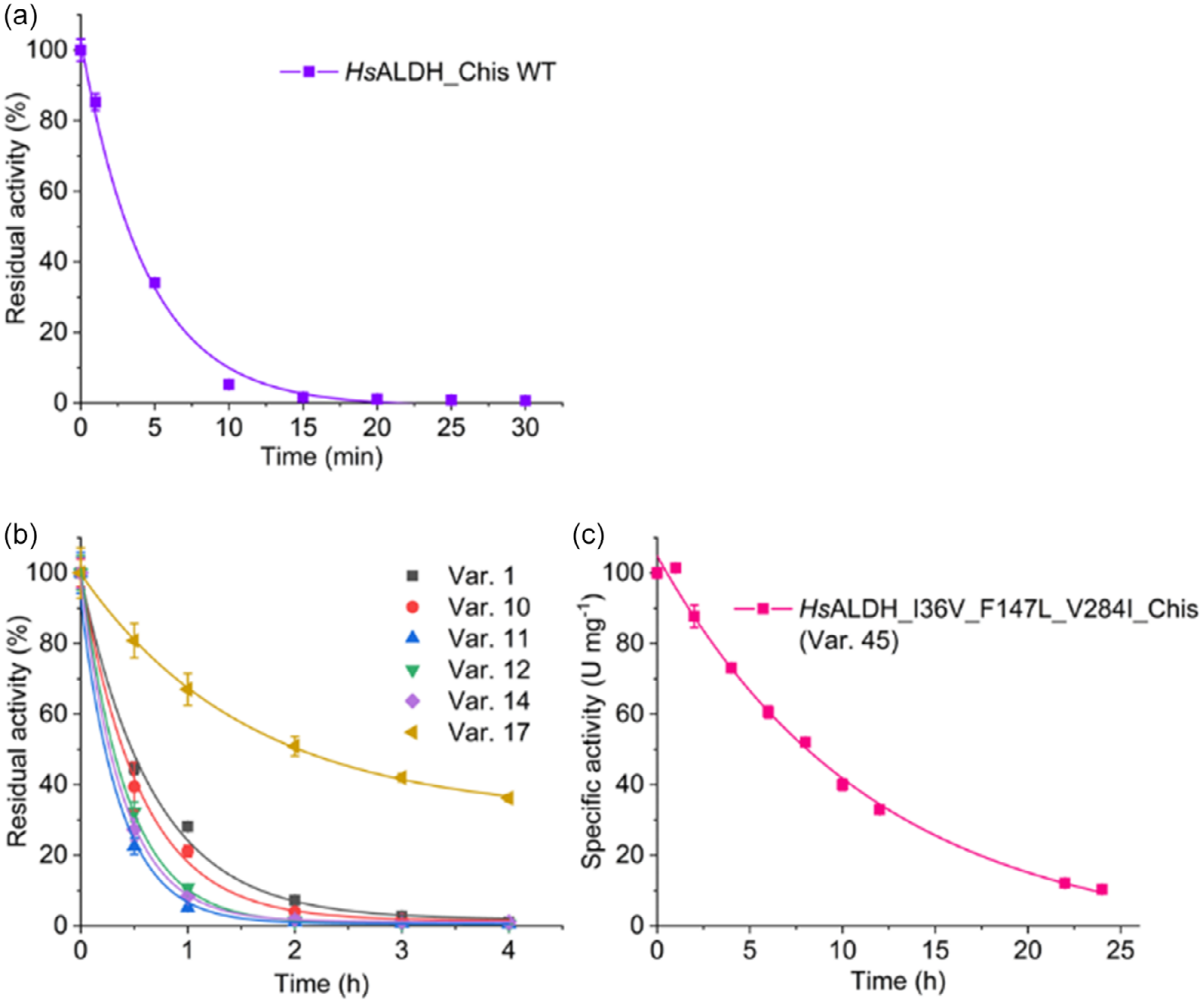
Optimization of enzyme stability and prolongation of the half‐life *t*_1/2_. a) Residual activities of wild‐type *Hs*ALDH_Chis, b) six variants selected from the second round of 96‐well plate screening, and c) *Hs*ALDH_I36V_F147L_V284I_Chis were monitored at 50 °C in triplicates (*n* = 3). Error bars indicate SDs. Activities were measured with 5 mm d‐glyceraldehyde and 5 mm NAD^+^ in 100 mm HEPES, pH 7.5, at 50 °C by withdrawing enzyme samples after incubation at 50 °C at defined time points (see also Table S6, Supporting Information).

Our recent study on the discovery and development of NAD^+^‐dependent ALDH for the oxidation of d‐glyceraldehyde to d‐glycerate identified GADH as one of the key enzymes in the cell‐free artificial minimal enzyme cascade.^[8c]^ The implementation of ALDH variants with optimized activity and substrate selectivity dramatically increased ethanol production in vitro and validated their efficacy in the cascade. However, a compromise between enzyme thermostability and cascade reaction temperature was indispensable. In that study, an ALDH from *Herbaspirillum seropedicae Z67* exhibited the highest activity (*k*_cat_ of 67.3 s^−1^) among the ALDH homologs characterized in two rounds of genome mining, but showed a half‐life of ≈3 min at 50 °C (**Figure** **1**a). Since the initial conceptualization of cell‐free minimal enzyme cascades, the reaction temperature of 50 °C has been set according to the use of several thermostable enzymes, the prevention of microbial contamination as well as better separation of target products like isobutanol. Taking advantage of our recently customized AADS for UHTS, the present work aims to improve the (thermos‐)stability of *Hs*ALDH without compromising its intrinsically high activity toward d‐glyceraldehyde. It should be an important step toward the establishment of a robust isobutanol cascade, which is our long‐term goal.^[8a]^

Randomized error‐prone polymerase chain reaction (epPCR) libraries were generated using the *Hs*ALDH variant, *Hs*ALDH_V284I_Chis, as a template. Its generation by residue swapping from the homolog, *Vp*ALDH (*Variovorax paradoxus* EPS), and enzymatic properties have been recently described.^[8c]^ Briefly, a more than fivefold and more than twofold increase in half‐life (*t*_1/2_) and turnover over 3 h, respectively, was observed at 45 °C. To investigate an appropriate mutation frequency for generating *Hs*ALDH mutant variants for effective screening, five libraries with varying mutation rates were generated (Figure S1, Supporting Information). The activity landscape analysis of five libraries on each 96‐well plate showed that the libraries were theoretically completely inactive when the MnCl_2_ concentration used for epPCR was 0.5 mm and higher (Table S1, Supporting Information). The library generated with 0.25 mm MnCl_2_ resulted in 13.4% variants with more than 80% activity of the template, a reasonable compromise to obtain a high variability of the library while maintaining a high probability of finding active (potentially optimized) variants. According to sequence analysis of randomly selected clones, the library contains an average of four amino acid substitutions. The optimized transformation efficiency resulted in a library complexity of ≈63,000 variants (Table S2, Supporting Information).

**Figure 2:**
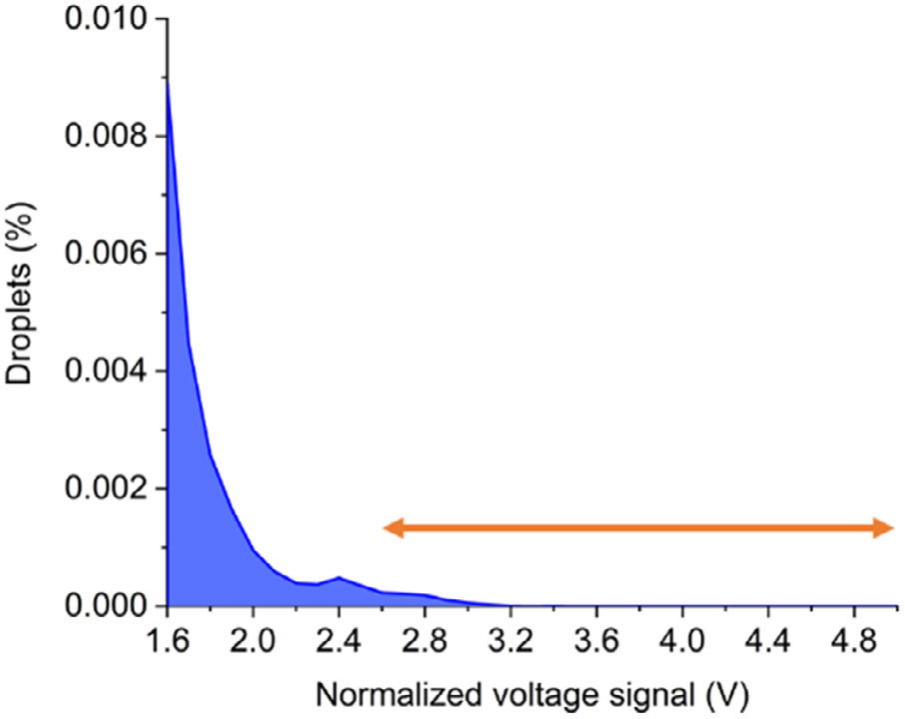
Histogram with normalized voltage signal derived from AADS screening of the *Hs*ALDH_Chis library. Droplets above the threshold of 2.6 V were collected.

An AADS pretest was performed using the *Hs*ALDH_V284I_Chis template to analyze the distribution of droplet signals after encapsulation of single cells (*λ* = 0.1) (Figure S2, Supporting Information). When the droplet incubation was performed at 30 °C, a small shoulder appeared to the left of the main empty droplet peak after 4 h of incubation (green distribution curve in Figure S2a, Supporting Information), indicating the presence of a droplet population containing active enzymes. Droplets with higher activity of encapsulated enzymes, registered further to the left,^[1a]^ remained small after 23 h of incubation (red distribution curve in Figure S2a, Supporting Information), indicating that a sufficient total turnover number (TTN) has not been achieved at this temperature. In addition, the overall transmittance decreases with time and the peak after 23 h of incubation is shifted to the far left, indicating that further extension of the incubation time will not optimize signal resolution. At an increased incubation temperature of 40 °C, a distinct second peak appeared when droplets were incubated for 1.5 to 4.0 h (blue and green distribution curves in Figure S2b, Supporting Information). At 50 °C, the peaks are more broadly distributed and the shoulder structures are almost unrecognizable (0 to 23 h; Figure S2c, Supporting Information). The temperature of 50 °C appears to be too high to achieve sufficient TTN before complete enzyme inactivation. In addition, the background development was significantly faster. Considering enzyme inactivation and background signal, an optimal temperature should be around 40 °C to achieve sufficient TTN and good signal resolution between inactive/empty droplets and active variants. While separation between active and empty droplets was achieved, the difficulty arose from the faster development of the background signal in the d‐glyceraldehyde assay compared to other enzymatic assays, likely due to unspecific reactions of d‐glyceraldehyde with cell lysate components. The conditions for droplet incubation were further refined by monitoring the residual activity of the template variant and mutant library in a temperature gradient (Figure S3, Supporting Information). The greatest effect was observed at 45.5 °C, the crossover point of the TTN of the template variant and the mutant library. Part of the library population retains a higher residual activity than the template variant. Further monitoring of the inactivation time courses confirmed that 45 °C is an appropriate temperature to heat challenge the library prior to screening (Figure S4, Supporting Information). Based on these observations, cells containing the epPCR library were encapsulated according to the Poisson distribution with an expectation of *λ* = 0.1, incubated for 6 h at 45 °C for reaction, and 1.3 × 10^6^ droplets were screened (**Figure** **2**, Table S3, Supporting Information). 1072 droplet hits were obtained.

After DNA recovery, a small fraction of the resulting clones was first analyzed in a 96‐well plate (Figure S5, Supporting Information). Surprisingly, only 7% of the clones analyzed showed at least 80% of the activity of the template variant. A further 6% of the clones showed activity between 20% and 80% of the template variant and a further 9% showed less than 20% but detectable activity, i.e., merely a total subpopulation of 21% showed activity. Accordingly, accumulation of improved variants was not observed, as 13% of the initial library showed more than 80% activity. When five randomly selected negative clones were sequenced, four showed no significant aberration in the nucleotide sequence, but one showed a large deletion of ≈360 amino acids, corresponding to three quarters of the protein sequence.

Due to the Poisson distribution of cell encapsulation in droplets, ≈5% of the total number of droplets containing cells are expected to be multiply occupied (Table S4, Supporting Information). Considering that less than 10% of variants can be derived from multioccupied droplets and 90% from single‐occupied droplets, the percentage of inactive clones after DNA recovery is extremely high. There are many factors to consider: droplet variation due to cell and droplet preparation (e.g., protein expression), possible fluctuation of assay results, accuracy of signal detection and molecular cloning (although no difference in percentage of active clones was observed between different methods of library preparation, i.e., restriction cloning and MEGAWHOP), including the potential for unintended accumulation of certain DNA constructs/variants, proper droplet actuation at the sorting electrode, and correct droplet direction to the proper channel. However, the discovery of a significantly improved variant 1 (hereafter referred to as var. 1) from the test plate motivated us to perform further screening on 96‐well plates (Table S5, Supporting Information). Var. 1 showed not only a moderate improvement in activity but also a significant increase in thermostability, retaining 45% of the initial activity after 30 min of incubation at 50 °C. In contrast, the wild‐type form or the template variant of *Hs*ALDH almost completely lost its activity. The high thermostability of var. 1 was also reflected in its *t*_1/2_, with a gain of almost three degrees Celsius.

**Figure 3:**
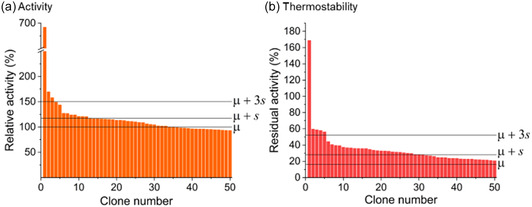
96‐well screening of the epPCR library. a) The activity and b) thermostability landscape of each of the top 50 clones is shown. (a) The mean activity of the template is marked with *μ* at 100%; *μ* + s and *μ* + 3*s* are 117% and 150%, respectively. (b) The mean residual activity of the template after 30 min of incubation at 50 °C is marked with a *μ* of 16%; *μ* + *s* and *μ* + 3 *s* are 28% and 52%, respectively.

In an attempt to accumulate optimized variants, the population of variants recovered from the AADS screening was subjected to two rounds of 96‐well screening. In the first round, three out of 3276 clones showed an improved initial activity of ≥150% (= *μ* + 3*s*) and a further 11 clones showed an activity of ≥117% (= *μ* + *s*) compared to the activity of the template (a 3*s* hit rate of 0.1% and a 1*s* hit rate of 0.4% for improved activity; **Figure** **3**a). A heat challenge was also performed on cell lysates from the most active 365 clones to analyze thermostability: five clones showed residual activity of ≥52% (= *μ* + 3*s*) and a further 25 clones showed residual activity above the lower threshold of 28% (= *μ *+ *s*) (a 3*s* hit rate of 0.2% and a 1*s* hit rate of 0.9% for improved thermostability; Figure 3b).

**Figure 4:**
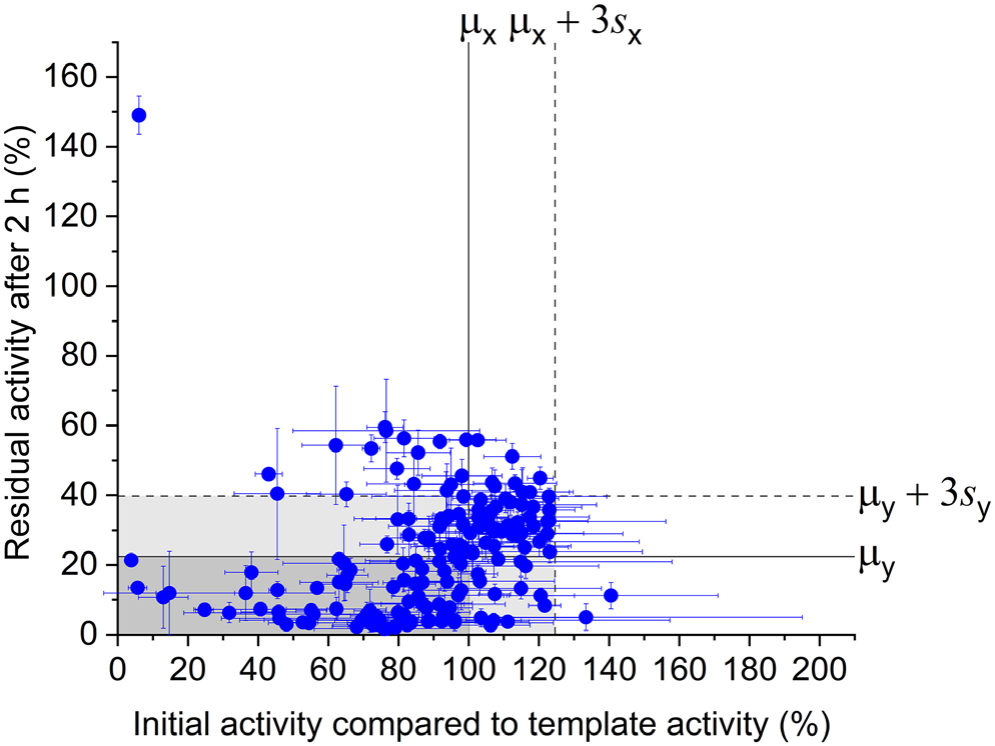
Second rescreening of the epPCR library. The second round of rescreening was performed with 168 selected clones in triplicates (*n* = 3). Their initial activities as a percentage of the template activity are plotted against their residual activities after 2 h of incubation at 50 °C (see Figure S7, Supporting Information for 1 h incubation). The mean activity of the template and the threshold for hits are shown as *μ*_*x*_ = 100% and *μ*_*x*_ + 3*s*_*x*_ = 125% for the first measurements at *t* = 0 h, *μ*_*y*_ = 22% and *μ*_*y*_ + 3*s*_*y*_ = 40% for the measurements at *t* = 2 h, respectively.

The second round of 96‐well plate screening was performed in triplicates (*n* = 3) with a total of 168 candidates from the first round, including 43 clones above the thresholds and a further 125 clones based on activity and thermostability profiles. The number of mutant variants was thus gradually restricted over two rounds of 96‐well plate screening and the loss of improved variants due to false negatives had to be avoided. Initial activity (Figure S6, Supporting Information) as well as residual activities after 1 h (Figure S7, Supporting Information) and 2 h of incubation (**Figure** **4**) at 50 °C were measured. Only two clones showed significantly higher initial activities than the *μ*_*x*_ + 3*s*_*x*_ threshold of 125% of the template enzyme (a 3*s* hit rate of 1.2% for improved activity; Figure S6, Supporting Information). However, a total of 59 clones showed initial activities higher than the template (1*s* hit rate of 36.3% for improved activity), and 25 clones still showed residual activities of ≥40% (= *μ*_*y*_ + 3*s*_*y*_) after 2 h of incubation (a 3*s* and 1*s* hit rate of 14.9% and 37.5%, respectively, for improved thermostability; Figure 4). No variants were found that were significantly superior in both activity and stability above *μ* + 3*s*, which should be in the upper right of the two‐parametric plot. However, 44 hits were found in the area above *μ*_*x*_ and *μ*_*y*_ (26% of the population with common medium improvements).

In summary, an accumulation of positive hits was successfully achieved in two rounds: the number of variants with a significant improvement in thermostability above 3*s* increased from five in the first round (Figure 3b) to 25 in the second round (15% of the population; Figure 4). The stricter heat challenge conditions and replicate measurements (only feasible for a limited number of variants) in the second round also ensured a high reliability of the screening results. In addition, var. 34 with a reduced initial activity of 6% and a 2 h activity of 149% (shown in the upper left of Figure 4) was noted, as its unusually high thermostability with presumed thermoactivation was repeatedly recognized during the screening campaign.

### 2.2 Mutation Analysis

To identify single mutations that confer advantages in activity and thermostability, 45 clones from the second round of 96‐well plate screening (including all variants with activities of ≥m + 2σ and/or thermostabilities of ≥m + 3σ) were sequenced and 34 unique variants were identified. The number of introduced amino acid mutations varied from one to five, with the most stable var. 34 having the highest number of mutations, five (Table S5, Supporting Information). The average number of mutations among the selected variants was ≈2, which is less than that observed in the initial library (average of four amino acid substitutions). Mutational hotspots (e.g., seven stabilizing mutations were found at positions 30 to 40) and flexible positions accepting multiple amino acid substitutions (e.g., I36V, I36F) were also detected. Interestingly, the mutation that reverts position 284 to the amino acid found in wild‐type *Hs*ALDH (I284V) was also observed (var. 15 in Table S5, Supporting Information), suggesting that a valine or isoleucine residue at this position makes a rather insignificant difference in single‐cell TTN at 45 °C; however, var. 15 also carries the activity enhancing A414T mutation.

First, 18 top variants from the 34 unique variants identified were expressed in a small scale for characterization (Figure 1b). Second, seven double mutants of *Hs*ALDH_V284I_Chis containing the additional mutations I36V, I36F (flexible position) or Y306F, A414T, E317D, I137F or R330C (selected for their occurrence in multiple variants (marked with colors in Table S5, Supporting Information) were generated for single mutation analysis (Table S5, Supporting Information). The combined information from these data can suggest interactions and epistasis of mutations in terms of activity and thermostability.^[^ ^10^ ^]^

Var. 1, 10–12, 14, and 17 (underlined in Table S5, Supporting Information), as well as additional double mutant variants, var. 35 and 36 (eight variants in total), were selected for further analysis because they exhibited higher activity than the template variant and greater putative thermostability as evidenced by residual activity after heat challenge and *T*_m_ values. The *t*_1/2_ of the double mutants ranged from 14 min to 2.2 h, a significant improvement from the 3 to 10 min of the wild type and the template variant (Table S6, Supporting Information). The most thermostable variant was var. 35, containing the I36V substitution, with a *t*_1/2_ of 2.2 h (2^5^‐fold improvement over the wild‐type enzyme) and a *T*_m_ of 58.3 °C (+8.8 °C above the wild type). *Hs*ALDH_I36V_V284I_Chis was therefore used as the next template to incorporate the third mutation. The following mutations were tested for compatibility and possible synergistic effects with the two existing mutations:^[10a]^ K221R, I434T, I137F, A414T, Y306F, F64Y, K415M, L432M, F147L, A208T, and Q334R (marked with * in Table S5, Supporting Information). More specifically, the selection of mutations A414T, Y306F, and L432M for triple mutants was based on their potential advantage in activity (especially at the lower substrate concentration, suggesting an improved *K*_m_ value). The K221R, I434T, F64Y, and K415M mutations were selected for their thermostability advantage. The I137F mutation seems to be beneficial for both thermostability and activity. Mutation R330C was also further analyzed due to its multiple occurrences and small contribution to thermostability. In addition, mutations F147L and A208T from the superthermostable var. 34 were analyzed as potential mutations causing the increase in thermostability, and the possibility of retaining activity while achieving the same stabilizing effect was sought (var. 34 with 1.8 U mL^−1^ compared to the template variant with 29.3 U mL^−1^). Most of the 11 triple mutants generated (Table S6, Supporting Information), except for var. 44, 45, and 47, retained high activities above 30 U mg^−1^ against 5 mm d‐glyceraldehyde at 50 °C. In particular, var. 41 was the most active with 36 U mg^−1^, improving the activity of the wild‐type enzyme by 52% and that of the template by 86% (Table S6, Supporting Information). Its *t*_1/2_ was slightly decreased to 1.9 h (Table S6, Supporting Information). The highest *T*_m_ of 62.0 °C was recorded for var. 37, which is 12.5 °C higher than the *T*_m_ of wild‐type *Hs*ALDH. The *t*_1/2_ of this variant was improved to 3.5 h (2^6^‐fold improvement over the wild‐type enzyme). Var. 45, containing F147L from the super‐thermostable mutant var. 34, exhibited a similarly high *T*_m_ of 61.0 °C, but the effect of this mutation on the *t*_1/2_ was more dramatic, reaching 8.1 h; a more than 2^7^‐fold improvement over the wild type form and almost 2^2^‐fold improvement over the double mutant. In addition, substrate screening (Table S7, Supporting Information) confirmed that the activity of the mutants toward 5 mm isobutyraldehyde (a possible side reaction in the isobutanol cascade)[^8^, ^11^] remained negligible with ≤3% of the rate measured for d‐glyceraldehyde. Furthermore, the most thermostable var. 45 retained the undesired conversion of 5 mm acetaldehyde (an important intermediate in the ethanol cascade)[^8^, ^11^] as low as the template variant, although many other variants recovered activity toward acetaldehyde (Table S7, Supporting Information). These favorable properties of var. 45 supported the selection of F147L as the third mutation to be incorporated (Figure 1c). Further **Purification Tag Engineering** (see Supporting Information) of this variant gave rise to *Hs*ALDH_I36V_F147L_V284I_GS‐linker_Nhis (referred to as StEP template below) with a further improved *t*_1/2_ of 12.7 h by retaining high activity.

### 2.3 In Vitro Recombination of Mutations

The second round of 96‐well screening yielded more than 30 unique variants carrying multiple mutations in their genes. This resulted in the identification of a total of 62 different amino acid exchanges. A more efficient strategy was needed to reduce the time and cost associated with the stepwise construction of an ultimate variant. Therefore, another focused library of selected mutations was generated based on in vitro DNA recombination. This method would allow efficient search across a diverse combinatorial repertoire of mutations at once, as well as efficient accumulation of beneficial mutations while removing deleterious ones, a significant advantage over performing multiple rounds of randomization.^[^ ^12^ ^]^ The staggered extension process (StEP), based on a modified PCR, is one of the most widely used in vitro recombination techniques.^[^ ^13^ ^]^ For this purpose, 11 mutations from the previous screening were identified as potentially beneficial to allow fast and easy screening with a small number of microtiter plates. Theoretically, 2048 combinatorial possibilities can be provided. The selected mutations included six mutations presented with Mutation 3 in Table S6, Supporting Information and another five mutations from Table S5, Supporting Information. They were selected based on their activity (L432M), stability (K415M, F64Y, K221R, I434T, A39T, A246T, Y190F), or both (V158A, P371L, I137F). Furthermore, the StEP procedure was refined in order to reduce potential biases during the recombination process^[13a]^ and to increase the proportion of full‐length progeny (see **StEP** in the Supporting Information).

After optimizing the expression conditions (Figure S8, Supporting Information) and confirming the bulk activity of the library (Table S10, Supporting Information), the StEP library was screened in a single 384‐well plate. Nine unique hits were identified by measuring initial and residual activity after incubation at 50 °C for 17 h (Table S11, Supporting Information). One spontaneous mutation was also identified (G11D mutation for variant 53 in Table S11, Supporting Information). Consistent with the observation in Table S6, Supporting Information, the most interesting mutation was K221R since variant 51 containing this single mutation showed its clear advantage in *t*_1/2_, which increased from 9.8 to 13.0 h (Table S11, Supporting Information). Its lysate activity at 5 mm substrate concentration was slightly reduced to ≈85%, but was twofold higher when normalized to cell density (Table S11, Supporting Information). This substitution between two positively charged amino acids was found in three of the nine hits. Compared to the best hit, variant 51, the others did not show a significant improvement in *t*_1/2_, except for variant 58 containing F64Y and V158A with a slight increase in *t*_1/2_ of about 1 h. While the fact that the best variant 51 contained only one additional mutation was a rather disappointing result for the laborious StEP procedure, other variants allowed to deduce correlations of single mutations. In particular, when comparing variants 52 and 58, the V158A mutation showed an apparent stabilizing effect (Table S11, Supporting Information): the introduction of F64Y in variant 52 significantly reduced the *t*_1/2_ by more than 1 h from 9.8 to 8.4 h. However, additional introduction of V158A, as shown in variant 58, restored or even increased the *t*_1/2_ to 10.8 h. Another potentially stabilizing mutation identified was L432M: as shown in variant 59, its introduction restored the *t*_1/2_ reduced by the A39T, I137F and Y190F mutations (see variants 54 and 57).

Focusing on the three mutations discussed above, K221R, V158A and L432M, the following five final variants were generated using the StEP template: I36V_F147L_**K221R**_V284I (FIN1); I36V_F147L_V284I_**L432M** (FIN2); I36V_F147L_**V158A**_**K221R**_V284I (FIN3); I36V_F147L_**K221R**_V284I_**L432M** (FIN4); I36V_F147L_**V158A**_**K221R**_V284I_**L432M** (FIN5). FIN3 and FIN4 increased the activity toward 5 mm d‐glyceraldehyde by 30–50% compared to the StEP template when measured in lysates (Figure S9b, Supporting Information). In contrast, the hexa‐mutant (FIN5) exhibited only 77% putative activity in lysate. However, given the significantly lower protein expression, its specific activity measured with a purified enzyme could potentially be higher than that of the StEP template (Figure S9, Supporting Information). FIN3 to FIN5 in the lysates also showed higher melting temperatures up to 63.2 °C compared to the template with a *T*_m_ of 60.5 °C (here, temperature increment of 0.5 °C per 1 min for thermofluor; Figure S10, Supporting Information). This was also the case when 3% v/v isobutanol was added: FIN3 showed a *T*_m_ of 53.7 °C while the template had a *T*_m_ of 50.2 °C.

**Figure 5:**
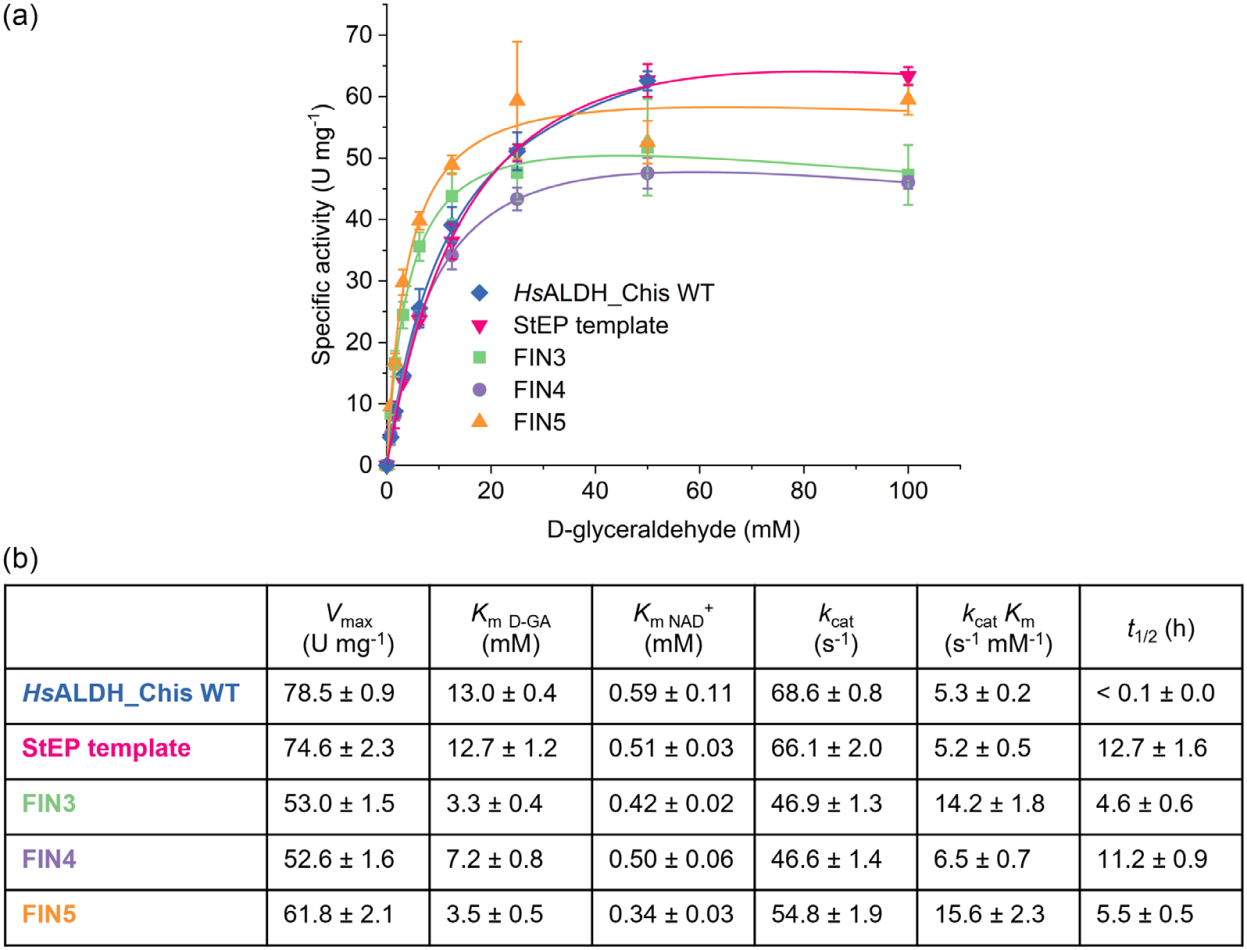
Kinetic characterization of the final *Hs*ALDH variants. a) Graphical representation of the catalytic rates of the variants, wild‐type and StEP template as a function of the substrate concentration. b) Kinetic parameters were estimated using the Michaelis–Menten equation. Measurements were performed with 5 mm d‐glyceraldehyde and 5 mm NAD^+^ in 100 mm HEPES, pH 7.5, at 50 °C in triplicates (*n* = 3). The wild‐type enzyme was analyzed at 30 °C due to its low thermostability. SD is shown as error bars. Kinetic characterization of the final *Hs*ALDH variants. (a) Graphical representation of the catalytic rates of the variants, wild‐type and StEP template as a function of the substrate concentration. (b) Kinetic parameters were estimated using the Michaelis–Menten equation. Measurements were performed with 5 mm d‐glyceraldehyde and 5 mm NAD^+^ in 100 mm HEPES, pH 7.5, at 50 °C in triplicates (*n* = 3). The wild‐type enzyme was analyzed at 30 °C due to its low thermostability. SD is shown as error bars.

**Figure 6:**
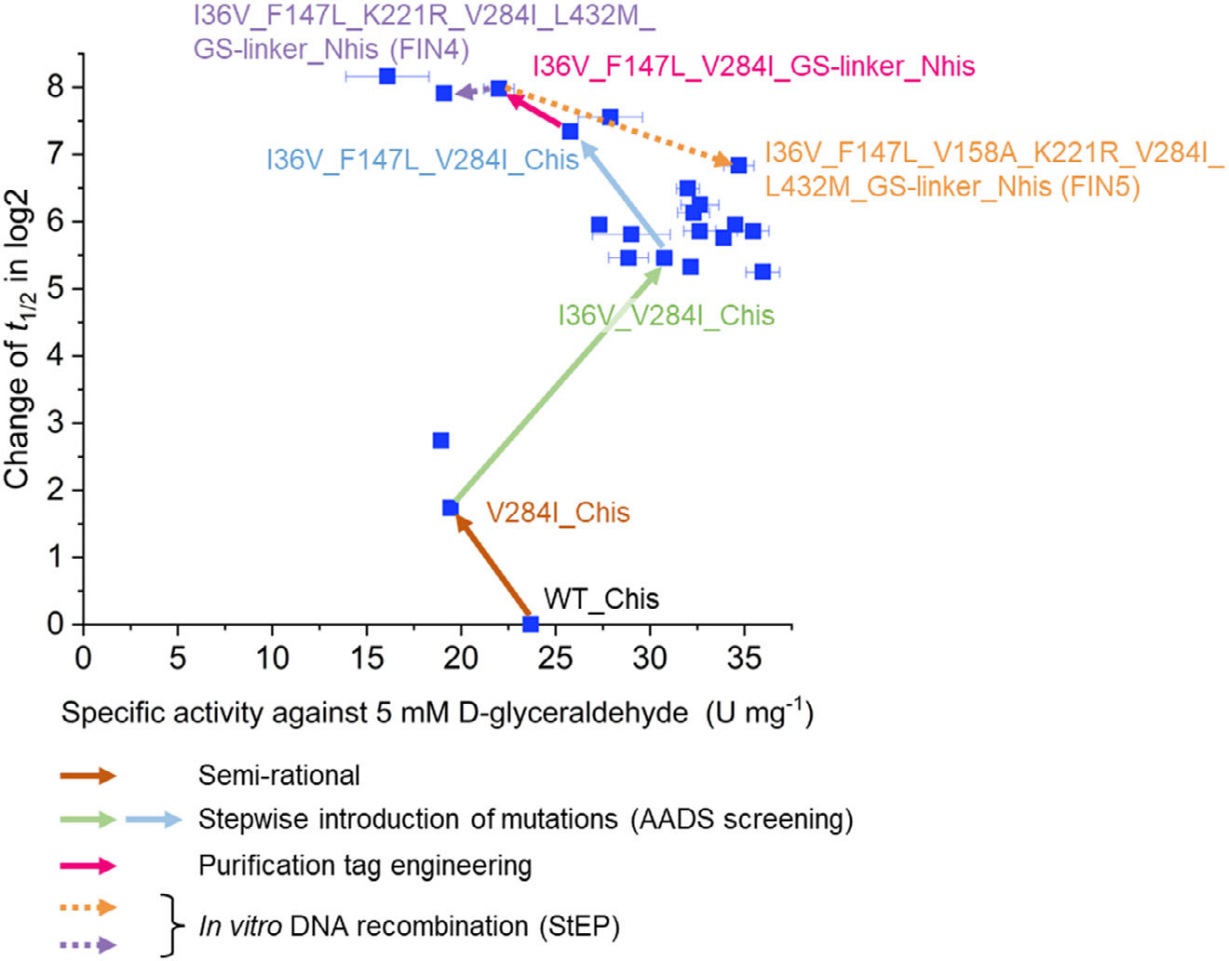
Pathway for the engineering of thermostable *Hs*ALDH variants. Representative *Hs*ALDH variants generated in each engineering step from the wild‐type form (WT_Chis) to the final variants, I36V_F147L_K221R_V284I_L432M_GS‐linker_Nhis (FIN4) and I36V_F147L_V158A_K221R_V284I_L432M_GS‐linker_Nhis (FIN5), are shown in a plot of specific activities measured with 5 mm d‐glyceraldehyde and 5 mm NAD^+^ in 100 mm HEPES, pH 7.5, at 50 °C in triplicates (*n* = 3; error bars indicate SD) versus half‐lives *t*_1/2_ at 50 °C. Pathway for the engineering of thermostable *Hs*ALDH variants. Representative *Hs*ALDH variants generated in each engineering step from the wild‐type form (WT_Chis) to the final variants, I36V_F147L_K221R_V284I_L432M_GS‐linker_Nhis (FIN4) and I36V_F147L_V158A_K221R_V284I_L432M_GS‐linker_Nhis (FIN5), are shown in a plot of specific activities measured with 5 mm d‐glyceraldehyde and 5 mm NAD^+^ in 100 mm HEPES, pH 7.5, at 50 °C in triplicates (*n* = 3; error bars indicate SD) versus half‐lives *t*_1/2_ at 50 °C.

Finally, three purified variants, FIN3–FIN5, were characterized in comparison with the previous variants. First, the modified NHis‐tagged “StEP template” and the CHis‐tagged wild‐type are very similar in their kinetic properties (**Figure** **5**). This suggests that the introduction of three mutations and the N‐terminal sequence engineering in the StEP template completely compensated the initial disadvantage of the NHis tag in activity. A decrease in *V*_max_ was observed for FIN3 to FIN5; however, the *K*_m_ values for both substrate and cofactor were improved, leading to an increase in catalytic efficiency (*k*_cat_ *K*_m_^−1^; Figure 5b). These characteristics would be advantageous for their applications in the multienzymatic cascades, where the putative concentrations of d‐glyceraldehyde and NAD^+^ should be low. In this regard, the hexa‐mutant, FIN5, was the most superior in kinetic properties among all the variants generated during the *Hs*ALDH engineering campaign. It increased the *k*_cat_ *K*_m_^−1^ threefold to 15.6 s^−1^ mm ^−1^ compared to the StEP template or wild‐type CHis. In addition, the *K*_m_ of this variant for NAD^+^ was improved by more than 40% to 0.34 mm compared to wild‐type CHis with a *K*_m_ of 0.59 mm. In terms of thermostability, the *t*_1/2_ value of FIN5 decreased to 5.5 ha, which is still significantly higher than all variants in Table S6, Supporting Information before the introduction of the F147L mutation, but represents a significant loss in *t*_1/2_ compared to the StEP template variant. Therefore, the kinetic advantages of FIN5 would be significant if cascade reactions can be completed in several hours. In contrast, FIN4 variant showed the least affected *t*_1/2_ value of 11.2 h and the difference to the template is in the range of deviation (Figure 5). **Figure** **6** provides a summary of the entire *Hs*ALDH engineering campaign, showing the changes of representative variants in activity and *t*_1/2_ measured under standard assay conditions.

### 2.4 Structure Analysis

The mutations introduced into the final variants of *Hs*ALDH were analyzed using protein structure models predicted by AlphaFold 3.^[^ ^14^ ^]^ Residue Phe147, the key position for the dramatic increase in thermostability, was found at the NAD^+^ binding site. Both Phe and Leu residues at this position could interact with the hexacycle of the adenosine moiety of an NAD^+^ molecule. The Gibbs free energy‐based analysis of the mutations using the computational tool DDMut^[3d]^ gave only negative results for the analyzed mutations. All analyzed single mutations and their combinations were predicted to be insignificant (e.g., V284I with −0.19 kcal mol^−1^) to destabilizing with V158A being the most destabilizing with −2.56 kcal mol^−1^ (the more negative the value, the more destabilizing). However, another tool, Dynamut,^[^ ^15^ ^]^ which assesses protein dynamics and resulting changes in vibrational entropy, predicted the K221R, V284I, and L432M mutations as slightly stabilizing with ΔΔG from +0.08 to 0.57 kcal mol^−1^. FireProt v2.0 predicted I36V as an alternative residue based on consensus; however, the integrated FoldX analysis predicted a slight destabilization with +0.25 kcal mol^−1^ (here, the more negative, the more stabilizing).^[^ ^16^ ^]^ Similar to Dynamut, the FoldX function of FireProt v2.0 showed an insignificant but slightly favorable result for L432M with −0.12 kcal mol^−1^. HotSpot Wizard v3.1^[^ ^17^ ^]^ did not detect any stability hotspots based on sequence consensus. Nevertheless, the multiple sequence alignment estimated the frequency of Val at position 36 to be 18.5%, higher than the occurrence of Ile at 5%. Furthermore, all analyzed mutations showed a high probability of functional preservation up to 88%, with the lowest probability of 60% for F147L.

**Figure 7:**
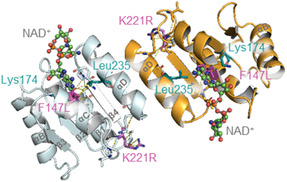
Rossmann folds of the dimerizing chain A and chain B. NAD^+^ molecules are bound to the sites. Identified mutations are highlighted in pink and key residues in blue.

A closer look at the protein models revealed further key features of the monomer and oligomer organization. Several candidate mutations were found at the Rossmann fold (**Figure** **7**). K221R is located on the loop between the αC and β4 and appears to play a significant role for the relative orientation of the β sheet and the overlying αC and αD through interactions with Val141, Pro143, Val219, Lys244, and Arg245 (predicted by DDMut).^[^ ^18^ ^]^ Interestingly, F147L, located on the β1 strand, might switch the interactions of the β sheet with the upper α helices: while the residue at this position on the β1 strand is responsible for the interactions between the neighboring β2 and β4 strands, Phe147 shows interactions with αC via Leu210, Leu214, and Leu215; however, the Leu147 mutation shows interactions with the αD via Leu235 (predicted by DDMut). Since chains A and B interact in a head‐to‐tail orientation, the antiparallel αD helices of both subunits in close proximity seem to play an important role, as does the F147L mutation (Figure 7). Similarly, L432M is located at the interface with another monomer, specifically on a β sheet at the monomer head (catalytic domain). This β strand interacts with another monomer at its C‐terminal β sheet on the tail oligomerization domain. The residue at position 432 on chain A lies adjacent to Val472 on chain B, the dimerization partner. Both Leu432 and Met432 show possible interactions with Val472. Surprisingly, the latter position corresponds to V475L of *Vp*ALDH,^[^ ^19^ ^]^ identified in another study on the engineering of a homolog, suggesting this interface as a possible hot spot regarding both enzyme activity and stability.

### 2.5 Temperature and Isobutanol Tolerance

The International Air Transport Association and the aviation industry have recognized that sustainable aviation fuels (SAFs) are essential to reducing the environmental impact of CO_2_ emissions for “net‐zero by 2050,” where SAFs could contribute ≈65% of the reduction in global emissions.^[^ ^20^ ^]^ Currently, only isobutanol and ethanol are approved as drop‐in biofuel precursors for alcohol‐to‐jet up to a maximum blend of 50%.^[20a]^ In particular, isobutanol has significant advantages over ethanol as an SAF precursor due to its lower hygroscopicity, lower vapor pressure, higher energy density, and similar physicochemical properties to gasoline.^[^ ^21^ ^]^ Gevo, Inc. has demonstrated the first transformation step toward large‐scale production of sustainable isobutanol from feedstock‐derived sugars using an engineered yeast strain at its 1.5 million gallon per year development facility in Luverne, Minnesota.[^20^, ^22^] However, the biotechnological production of isobutanol still faces several challenges, including high production costs and low production titers (a maximum of 50 g L^−1^ using in situ product removal has been reported to date).^[^ ^23^ ^]^ The main limitations of fermentation processes result from the physiological limits of cellular production systems and the high cytotoxicity of isobutanol;^[8a]^ ≥1% v/v isobutanol can already induce toxic effects (growth inhibition) on host strains such as *E. coli*.^[^ ^24^ ^]^ Postulating that cell‐free biosynthesis would circumvent this living cell‐associated problem of low solvent tolerance, our group previously investigated the influence of increasing isobutanol concentration on the productivity of the cascade and the stability of each enzyme.^[8a]^ The study demonstrated high stability of the ethanol cascade at ≤4% v/v isobutanol and identified ALDH as the least stable enzyme in the cascade, losing 50% of its activity immediately upon exposure to 3% v/v isobutanol.^[8a]^

**Figure 8:**
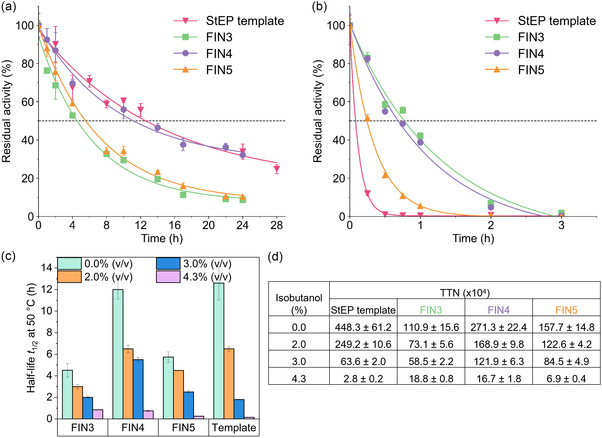
Stability of the engineered *Hs*ALDH generated from the StEP library. Residual activity of the variants was monitored at 50 °C in the a) absence of isobutanol and b) presence of 4.3% v/v isobutanol. Measurements were performed with 5 mm d‐glyceraldehyde and 5 mm NAD^+^ in 100 mm HEPES, pH 7.5, at 50 °C in triplicates (*n* = 3). SD is shown as error bars. Experiments at 2.0 and 3.0% v/v isobutanol are shown in Figure S11, Supporting Information. The decrease in half‐life with increasing isobutanol concentration from 0.0 to 4.3% v/v at 50 °C is shown in c). d) Theoretical TTN at different isobutanol concentrations were estimated according to Equation (1). Stability of the engineered *Hs*ALDH generated from the StEP library. Residual activity of the variants was monitored at 50 °C in the (a) absence of isobutanol and (b) presence of 4.3% v/v isobutanol. Measurements were performed with 5 mm d‐glyceraldehyde and 5 mm NAD^+^ in 100 mm HEPES, pH 7.5, at 50 °C in triplicates (*n* = 3). SD is shown as error bars. Experiments at 2.0 and 3.0% v/v isobutanol are shown in Figure S11, Supporting Information. The decrease in half‐life with increasing isobutanol concentration from 0.0 to 4.3% v/v at 50 °C is shown in (c). (d) Theoretical TTN at different isobutanol concentrations were estimated according to Equation (1).

Since a correlation between thermostability and organic solvent tolerance is frequently postulated, we wondered whether thermostabilization of our enzyme would also have a positive impact on its solvent stability.^[^ ^25^ ^]^ To this end, we tested the StEP template I36V_F147L_V284I_GS‐linker and three final variants (FIN3–FIN5) for stability at 50 °C in the presence of 0.0–4.3% v/v isobutanol (**Figure** **8** and Figure S11, Supporting Information). In the absence of isobutanol, four of the tested variants split into two types of time courses (Figure 8a). The StEP template and FIN4 showed higher stability with a *t*_1/2_ between 11 and 13 h. FIN3 and FIN5 had lower half‐lives of 4–6 h (i.e., ≈50% of the first two variants). All four variants, including the StEP template, significantly decreased in stability with each increase in isobutanol concentration (Figure 8c). In particular, the StEP template variant exhibited the highest susceptibility in isobutanol, despite its high thermostability. Specifically, its *t*_1/2_ at 2.0% v/v isobutanol decreased to 57% compared to that at 0.0% v/v isobutanol (Figure S11a,S12, Supporting Information). At 3.0% v/v isobutanol, the *t*_1/2_ further decreased to 15% and thus the StEP template dropped to the least stable variant with a *t*_1/2_ of 1.9 h (Figure S11b, S12, Supporting Information). In contrast, FIN3–FIN5 retained ≈50% of the initial stability at 3.0% v/v isobutanol (Figure S12, Supporting Information). At this isobutanol concentration, FIN4 indicates an almost doubled putative TTN of the StEP template, a completely inverted result of the TTN at 0.0% v/v isobutanol, where the StEP variant showed a 1.7‐fold higher TTN due to its advantageous *k*_cat_ (Figure 8d). At 4.3% v/v isobutanol, all variants were severely affected with a *t*_1/2_ of less than 1 h (Figure 8b). In particular, the StEP template was virtually inactive within 0.5 h with a *t*_1/2_ of 5 min. FIN3 and FIN4 were significantly more stable at 4.3% v/v isobutanol with *t*_1/2_ between 40 and 50 min. Their complete inactivation is expected after 3 h (Figure 8c). Throughout the range of 0.0–4.3% v/v isobutanol, FIN4 shows its advantages of high thermostability and isobutanol tolerance (Figure 8c), which would contribute to the highest TTN in cascade experiments targeting a final titer below 4.3% (Figure 8d).

In summary, this 50 °C isobutanol challenge clearly demonstrates the significant advantage of our final *Hs*ALDH variants in catalytic stability even in the presence of isobutanol. All four variants, the StEP template I36V_F147L_V284I_GS‐linker and FIN3–FIN5 required at least 1.9 h to inactivate the enzymes by 50% in the presence of 3% v/v isobutanol at 50 °C. FIN4 required as much as 5.0 h, a significant improvement over the ALDH variant from the initial study.[^8^, ^11^] Furthermore, all variants still displayed residual activity after 6 h (Figure S11b, Supporting Information). Although the correlation between the resistance to denaturation by temperature and organic solvents is complex, involving both internal (e.g., molecular mobility and stability) and external factors (e.g., protein hydration), this example confirms that the high thermostability gained by the *Hs*ALDH variants is also evident in the presence of isobutanol.^[^ ^25^ ^]^

### 2.6. Ethanol Cascade in the Presence of Isobutanol

To demonstrate the enhanced isobutanol tolerance of FIN4, it was incorporated into a cell‐free minimal cascade for ethanol production from d‐glucose in the presence of 4% isobutanol (Table S13, Supporting Information).^[8a,8c]^ The cascades were tested with 100 mm d‐glucose, from which a theoretical molar yield of 200 mm ethanol is expected upon full conversion. In this cascade, 50% of the ethanol originates from the direct conversion of 2‐keto‐3‐deoxy‐gluconate to pyruvate, while the remaining 50% is produced via the oxidation of d‐glyceraldehyde to d‐glycerate and subsequently to pyruvate (Figure S16, Supporting Information).^[8c]^ Therefore, achieving a yield exceeding 50% is a critical indicator that this pathway is working and the engineered ALDH variants are functional.

**Figure 9:**
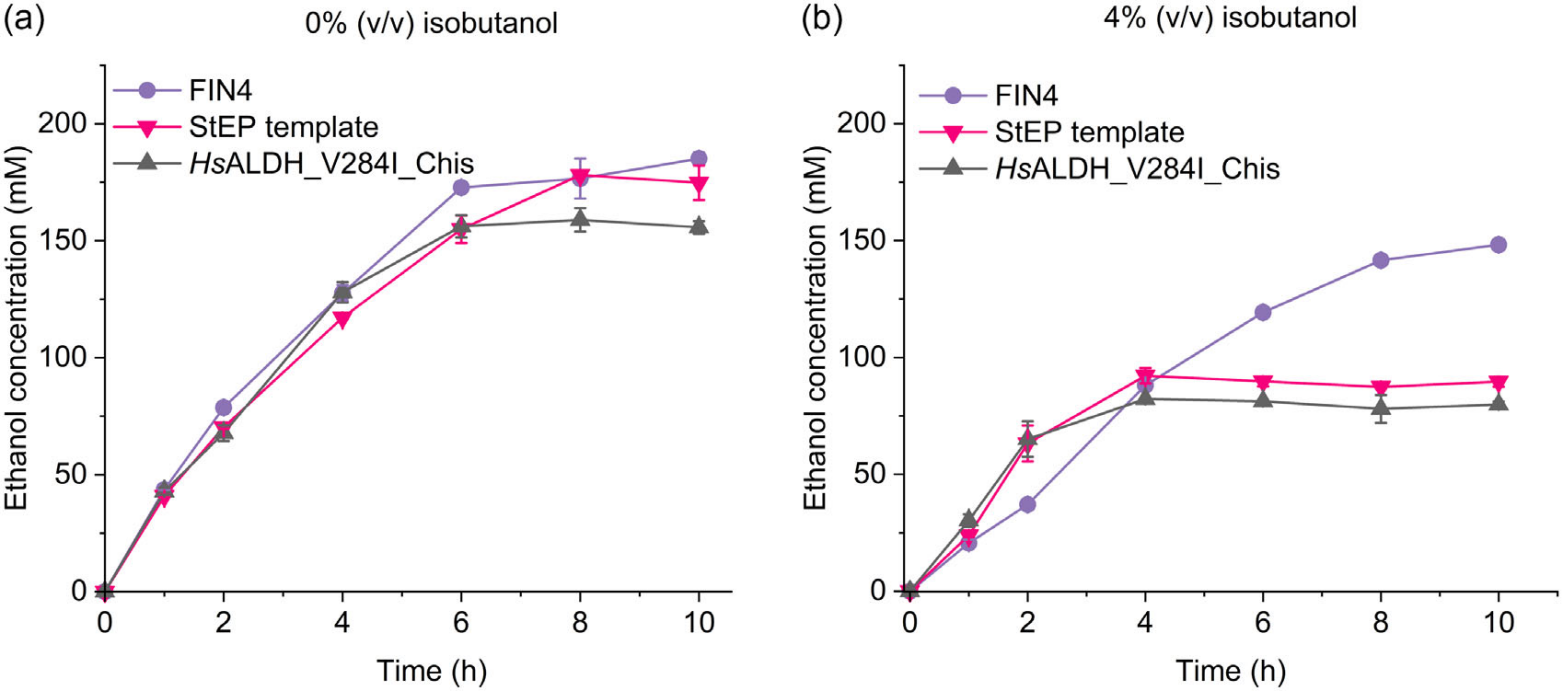
Ethanol production from d‐glucose via in vitro cascades using different *Hs*ALDH variants at 50 °C in the presence of a) 0% v/v and b) 4% v/v isobutanol. Cascade reactions were performed with 100 mm d‐glucose, 5 mm NAD^+^, 0.5 mm TPP, 5 mm MgCl_2_, and 200 mm HEPES, pH 8.0 in duplicates (*n* = 2). SD is shown as error bars. Ethanol production from d‐glucose via in vitro cascades using different *Hs*ALDH variants at 50 °C in the presence of (a) 0% v/v and (b) 4% v/v isobutanol. Cascade reactions were performed with 100 mm d‐glucose, 5 mm NAD^+^, 0.5 mm TPP, 5 mm MgCl_2_, and 200 mm HEPES, pH 8.0 in duplicates (*n* = 2). SD is shown as error bars.

In the absence of isobutanol, cascades with all three variants exhibited comparable ethanol yields and productivity at 50 °C. Cascade with FIN4 achieved a final yield of ≈185 mm (92.5%), while the StEP template and *Hs*ALDH_V284I_Chis cascades reached 178 mm (89%) and 158 mm (79%), respectively (**Figure** **9**a). This shows that all variants remain functional at 50 °C under isobutanol‐free conditions. The primary intermediate accumulating in these cascades was d‐glycerate, consistent with previous findings that identify the dehydration of d‐glycerate to pyruvate as the rate‐limiting step.^[8c]^ The maximum d‐glycerate concentration reaches up to 29 mm in the *Hs*ALDH_V284I_Chis cascade, 23 mm in the StEP template cascade and 19 mm in the FIN4 cascade, after which the concentration decreases following the completion of d‐glucose consumption (Figure S17–19, Supporting Information).

In the presence of 4% v/v isobutanol, the cascade with FIN4 demonstrated significantly higher ethanol production, achieving 148 mm (74%) ethanol after 10 h (Figure 9b). In contrast, the cascades with the StEP template and *Hs*ALDH_V284I_Chis produced only 92 mm (46%) and 82 mm (41%) ethanol, respectively, after 4 h, with no further increase observed (Figure 9b). This confirms that FIN4 is far more stable in the presence of 4% v/v isobutanol as evidenced by the ethanol yield exceeding over 50%, confirming that ALDH remains functional. Conversely, the StEP template and *Hs*ALDH_V284I_Chis cascades with less than 100 mm (50%) ethanol yield indicate their instability in 4% v/v isobutanol. Interestingly, these cascades exhibited faster initial ethanol production, likely due to the rapid inactivation of the StEP template and *Hs*ALDH_V284I_Chis variants, which reduces competition for NAD^+^ with GDH, leading to more efficient glucose consumption and accelerated ethanol production via the direct pathway (Figure S18,S19, Supporting Information).

Additionally, the StEP template and *Hs*ALDH_V284I_Chis cascades with 4% v/v isobutanol exhibited significant accumulation of d‐glyceraldehyde instead of d‐glycerate, reaching concentrations of 23 and 35 mm, respectively (Figure S18,S19, Supporting Information). In contrast, the FIN4 cascade maintained d‐glycerate as the primary accumulated intermediate, with d‐glyceraldehyde concentration peaking at only 5 mm after 4 h before being fully consumed. This provides further evidence of FIN4's superior stability and functionality in the presence of 4% v/v isobutanol. Together, these findings highlight the success of the engineering efforts to enhance stability and confirm that ALDH is no longer a bottleneck in the future isobutanol cascade.

## 3 Conclusion

Our recent study demonstrated the semirational approach to improve the substrate selectivity of ALDH homologs more than 20‐fold in favor of d‐glyceraldehyde over acetaldehyde.^[8c]^ An engineered GADH allowed to dramatically increase the ethanol production from 2.1 to 39 g L^−1^ with a linear productivity up to 99% yield.^[8b]^ However, this previous work also pointed out the disadvantage of GADH in regard of its low thermostability. Prompted by this challenge, the current work aimed to engineer GADH for improved stability, making it more suitable for use for the production of isobutanol. A directed evolution approach was performed in combination with microfluidic ultrahigh‐throughput and microtiter plate screening. The screening of a 63,000‐membered large library from gene‐wide randomization was considered more promising than a (semi‐)rational approach, as stability engineering can be more diverse and not limited to specific regions (e.g., substrate binding site or conserved sequence motifs). By selecting a mutation frequency of four amino acids on average, we obtained 34 putative hits containing 62 different amino acid exchanges, providing not only a wealth of information on beneficial and deleterious mutations and their functional relationships, but also a challenge to cope with the large number.

The AADS technology has demonstrated its advantage in high throughput (remarkable progress has been achieved in recent years),^[^ ^19^, ^26^ ^]^ the possibility to cover the complexity of the whole library and the miniaturization of the assay. On the other hand, the accumulation of improved variants is still one of the major challenges for AADS technology to verify its high potential in enzyme engineering. Reliable procedures for sample preparation and downstream processing, including DNA recovery, as well as improved signal resolution need to be established, although several difficulties arise from the microfluidics‐specific problem of Poisson distribution of droplets for cell encapsulation.

Stepwise introduction of three mutations identified from the screening results generated *Hs*ALDH_I36V_F147L_V284I_Chis with a *t*_1/2_ of 8.1 h, dramatically improving the wild type *t*_1/2_ of 3 min. Relocation and engineering of the hexahistidine tag sequence further improved the half‐life to 12.6 h (≈2^8^‐fold (i.e., 250‐fold) improvement) while maintaining the high turnover rate, *k*_cat_, of 66.1 s^−1^. Considering the critical temperatures provided as *T*_m_ values, the selection of the standard assay temperature of 50 °C based on the future cascade setup was also effective in observing the largest effects of the introduced mutations. The introduction of the F147L mutation contributed to overcome this critical point for the structural instability of the *Hs*ALDH variant. The StEP‐based approach using four designer DNA templates facilitated efficient screening and analysis of the combinatorial possibilities of beneficial mutations with reduced recombination bias. A hexamutant variant with moderate thermostability was found to be kinetically superior, with a threefold improvement in catalytic efficiency and a reduction in the *K*_m_ for the cofactor to less than 60% of the wild‐type enzyme—desirable characteristics for its implementation in a cell‐free cascade to effectively prevent reactive d‐glyceraldehyde accumulation and to better compete with other NAD^+^‐dependent enzymes.^[8a]^ Another variant with five mutations showed significantly higher isobutanol tolerance, retaining 50% of its initial activity after 6 h of incubation at 3% v/v isobutanol and 50 °C. Its application in the cell‐free ethanol cascade in the presence of 4% v/v isobutanol validated its superior stability and functionality. In conclusion, the beneficial mutations identified in this study seem to be unattainable by a solely computational approach and the *Hs*ALDH engineering presented in this work would represent a first step toward the future goal of realizing efficient isobutanol production in a cell‐free biocatalytic system, as ALDH has previously been identified as the least stable enzyme in the isobutanol cascade.^[8a]^ However, compared to the ethanol cascade, the more extensive isobutanol cascade presents additional challenges due to several bottleneck enzymes contained in the lower part of the cascade. In particular, reducing potential side reactions within the cascade is the major challenge for the next step. In combination with this, the cell‐free biosynthesis of isobutanol would greatly benefit from the high stability and catalytic performance of an engineered *Hs*ALDH. The ultimate goal would be a continuous production within a two‐phase isobutanol/water system for an economical downstream process, which requires further improvement of the produced isobutanol concentration.[^8^, ^27^]

## 4 Experimental Sections

4.1

4.1.1

##### Reagents

All chemicals used in this work were from Carl Roth (Karlsruhe, Germany), Thermo Fisher Scientific (Waltham, MA, USA), Merck (Darmstadt, Germany), Millipore (Darmstadt, Germany), New England Biolabs (NEB) (Frankfurt am Main, Germany), Promega (Walldorf, Germany), Qiagen (Hilden, Germany), Serva (Heidelberg, Germany), Sigma–Aldrich (Darmstadt, Germany), and VWR (Darmstadt, Germany). Oligonucleotides were purchased from Eurofins Genomics (Ebersberg, Germany) and full‐length genes with optimized codon usages were synthesized by ATG:biosynthetics GmbH (Merzhausen, Germany).

##### Strains and Plasmids

*E. coli* XL1‐Blue cells (*recA1 endA1 gyrA96 thi‐1 hsdR17 supE44 relA1 lac [F′ proABlacIqZΔM15 Tn10 (Tetr)]*) and NEB Turbo cells (*F′ proA+B+ lacIq ΔlacZM15/fhuA2 Δ(lac‐proAB) glnV galK16 galE15 R(zgb‐210:Tn10)TetS endA1 thi‐1 Δ(hsdS‐mcrB)5*) were purchased from Stratagene (Waldbronn, Germany) and NEB, respectively, and used for molecular cloning. *E. coli* BL21(DE3) cells (*F– ompT gal dcm lon hsdSB(rB‐ mB‐) λ(DE3 [lacI lacUV5‐T7 gene 1 ind1 sam7 nin5])*) from Novagen (Nottingham, UK) were used for recombinant protein expression. *H. seropedicae Z67* (DSM 6445) was purchased from DSMZ German Collection of Microorganisms and Cell Cultures GmbH (Braunschweig, Germany) for the extraction of genomic DNA (gDNA). *pET24a_HsALDH_V284I_Chis*, the template plasmid for epPCR library generation, was generated as described previously.^[8c]^ pET24a and pET28a cloning vectors were purchased from Novagen.

##### Library Generation

Randomized libraries were generated by epPCR using pET24a_*Hs*ALDH_V284I_Chis as a template.^[^ ^28^ ^]^ The whole plasmid construct was generated by the MEGAWHOP method (megaprimer PCR of whole plasmid).^[^ ^29^ ^]^ The procedure of epPCR and MEGAWHOP has been described previously.^[^ ^19^ ^]^ After drop‐dialysis of the DNA solution against dH_2_O for 1 h on a 0.025 μm VSWP membrane (Millipore), electrocompetent *E. coli* XL‐1 Blue cells prepared according to the protocol of NEB^[^ ^30^ ^]^ were transformed by electroporation with 4 ng of dialyzed DNA per 1 μL of the competent cells (Bio‐Rad MicroPulser Ec2 program at 2.5 kV). DNA isolated from the cloning strain was further purified by butanol precipitation prior to transformation of the expression strain *E. coli* BL21(DE3).^[^ ^31^ ^]^ Transformed cells were recovered in SOC medium^[^ ^32^ ^]^ for 1 h and a small portion of the cell suspension was then spread on LB (Luria‐Bertani) agar plates with 100 μg mL^−1^ kanamycin to estimate the transformation efficiency. The remaining cell suspension was transferred to 100 mL of LB broth with 100 μg mL^−1^ kanamycin in a baffled flask and incubated at 37 °C and 160 rpm (Heraeus and MaxQ 2000, Thermo Scientific) for 16 h. This preculture was used directly for *protein expression*.

##### Protein Expression

To prepare the library for microfluidic screening, 100 mL of the main culture (20% v/v ZYP‐5052 autoinduction medium^[^ ^33^ ^]^ or Terrific Broth (TB medium)^[^ ^34^ ^]^ in a baffled flask) with 100 μg mL^−1^ kanamycin was inoculated with the preculture at an OD_600_ of 0.07 and shaken at 37 °C and 160 rpm (Heraeus and MaxQ 2000, Thermo Scientific) until an OD_600_ of 0.5–0.6 was reached. By use of TB medium, protein expression was induced by the addition of 0.4 mm isopropyl‐β‐d‐1‐thiogalactopyranoside (IPTG). The temperature was decreased to room temperature for protein expression for 20 h. Cells were centrifuged at 3000 g for 20 min at 4 °C (Heraeus Fresco 21 centrifuge, Thermo Scientific) in 1.0 mL aliquots in 1.5 mL reaction tubes (Eppendorf). Supernatants were discarded, and the cell pellets were frozen at −20 °C until further use.

Heterologous expression of individual enzyme variants was performed in a similar manner. Cells were harvested by centrifugation at 4 °C and 3000 g for 30 min (Sorvall LYNX 6000, Thermo Scientific), the supernatant was discarded, and the cell pellet was stored in a 50 mL Falcon tube at −20 °C until protein purification. Protein purification was performed as previously described.^[8c]^

##### Analysis of Activity Landscapes and Mutation Frequency

*Hs*ALDH libraries generated with different MnCl_2_ concentrations of 1.00, 0.50, 0.25, 0.13, and 0.06 mm were each analyzed in a 96‐well deep well plate (Brand) to obtain activity landscapes. Transfer of media and other liquids was performed using the Freedom Evo 200 system (Tecan). Deep well plates filled with 1.2 mL LB broth containing 100 μg mL^−1^ kanamycin were inoculated with single colonies of transformed *E. coli* BL21(DE3) cells for protein expression. Wild‐type enzyme was sampled in six wells of each plate as a positive control. Empty vector controls (BL21(DE3) transformed with pET24a) and media controls were also included in three wells each as negative controls. The inoculated plates were covered with cell culture sheets and incubated at 37 °C and 1000 rpm (Microtiterplate shaker TiMix 5, Edmund Bühler) for 20 h. 15 μL of the precultures was transferred to the main cultures of 1 mL TB broth containing 100 μg mL^−1^ kanamycin in deep well plates. The plates were covered with cell culture sheets and incubated at 37 °C and 1000 rpm for 2 h. Backups (e.g., for plasmid isolation and DNA sequencing) were also prepared with 150 μL of 50% v/v glycerol and 88 μL of the preculture in 96‐well plates, checked for OD_600_, and frozen at −80 °C. Main culture plates were incubated at 37 °C for 2 h for growth and protein expression was induced by the addition of 0.4 mm IPTG. Cultures were further incubated at room temperature for 20 h. At the end of protein expression, final cell densities were measured by OD_600_ using 12.5 μL of culture diluted with dH_2_O in a total volume of 200 μL. Cells were harvested by centrifugation at 1400 g for 30 min at 4 °C (Rotanta 46 RSC Robotic, Hettich) in deep well plates and the supernatant was discarded. Cell lysis was performed by adding 500 μL of lysis buffer (2 mg mL^−1^ lysozyme, 0.1 mg mL^−1^ DNase I, 0.5 mg mL^−1^ polymyxin B sulfate, 100 mm HEPES pH 7.5) to the cell pellets. The lysis buffer with cells was shaken vigorously at 1400 rpm (Microtiterplate shaker TiMix 5, Edmund Bühler) at 37 °C for 1.5 h. The plates were centrifuged as described above and 300 μL of the supernatants was transferred to 96‐well plates for enzyme activity assays.

##### ALDH Activity Assay

The activity and kinetic properties of aldehyde dehydrogenases were determined photometrically as previously described.^[8c]^ Cell lysates were diluted 1:2500 or 1:5000 for measurement in a total volume of 100 μL on a 96‐well plate. For heat challenge, cell lysates were transferred to PCR plates and covered with a silicone cover for incubation in a PCR cycler at 50 °C. Subsequently, residual activities were determined.

##### Determination of DNA and Protein Concentrations

DNA and protein concentrations were determined at *λ* = 260 nm and *λ* = 280 nm, respectively, using a NanoPhotometer P‐Class (Implen). Molecular weights (MW) and molar extinction coefficients (*ε*_280_) of the enzyme variants were determined using the ProtParam tool (Expasy).^[^ ^35^ ^]^

##### Microfluidic Screening

The following three solutions were prepared for droplet production: oil solution (1.5% Picosurf in Novec 7500), substrate and lysis solution (5 mm d‐glyceraldehyde, 5 mm NAD^+^, 5 mm WST‐1, 2.5 mm tartrazine, 20 μg mL^−1^ mPMS, 4% v/v CelLytic B, 120 kU mL^−1^ rLysozyme in 100 mm HEPES, pH 7.5), and cell solution (cells with an OD of 0.0015 (*λ* = 0.1), 25% Percoll in 100 mm HEPES, pH 7.5). These were loaded into gas‐tight syringes (SGE) and mounted in a syringe pump (Flow‐Start Evo, Future Chemistry). Droplets were generated as previously described and collected in a self‐made droplet chamber.^[^ ^36^ ^]^ Approximately 0.5 mL of oil was released from the chamber to prevent overpressure during droplet incubation at elevated temperatures. Both tube ends were sealed by heating with a lighter and crimped with tweezers. The generated droplets were incubated for 1 h at room temperature to allow for complete lysis of the cells and release of the enzymes into the droplet space. The droplet chamber was then placed in an incubator at 45 °C for 6 h. Droplets were reinjected into a measurement chip and enzyme activity within the droplets was measured using the cofactor‐coupled WST‐1 assay with an absorbance‐activated droplet sorter (https://openwetware.org/wiki/DropBase:AADS)^[1a]^ Droplets reaching a user‐defined threshold were dielectrophoretically sorted and collected in a vial. Droplets were counted and their corresponding peak value was tracked using a customized LabVIEW program.

##### DNA Recovery

The collected droplets were de‐emusified, and DNA from the sorted variants was extracted as previously described.^[36a]^ Extracted DNA was further purified using NucleoSpin Gel and PCR Clean‐up Mini Kit (Macherey‐Nagel) and eluted with 50 μL dH_2_O. The gene of interest of the sorted variants was amplified using 1x Accuzyme polymerase mix (Bioline), 21 μL of the purified DNA, 3% v/v DMSO, and 0.25 μm of each of forward and reverse primers in a total volume of 50 μL. Two reaction mixtures were prepared. The PCR program was set to 98 °C for 3 min, 46 cycles of 98 °C for 30 s, 61 °C for 30 s, and 72 °C for 3.5 min, followed by 72 °C for 5 min and cooling to 4 °C. Approximately 2.1 μg of DNA was obtained after agarose gel (2% w/v) separation and extraction. The purified PCR product was subsequently used for MEGAWHOP for 31 cycles and elongation for 13:45 min. DpnI digestion was performed overnight and the amplified DNA containing putative hits was further purified by drop‐dialysis and butanol precipitation prior to electroporation of XL1‐Blue.

##### Generation and Screening of StEP Library

Four template plasmids for StEP PCR were designed according to Figure S13, Supporting Information and synthesized using codon optimization (ATG: biosynthetics GmbH). Repetitive DNA sequence patterns were modified in the codons. The template gene was prepared by digestion with SphI and PsiI followed by separation and purification on agarose gel. StEP PCR mixtures (100 μL divided into eight PCR tubes) contained 0.15 pmol of the purified DNA fragment, 30 pmol of each forward and reverse primer, 3% v/v DMSO, and 1x Accuzyme polymerase mix. The PCR programs for StEP PCR 1 and 2 are shown in Table S9, Supporting Information. The PCR products were purified on an agarose gel, and 2 ng μL^−1^ of the extracted target DNA fragment was subjected to the second PCR (nested PCR)^[^ ^37^ ^]^ using 1x Accuzyme polymerase mix, 3% v/v DMSO and 0.5 μm of each T7 forward and reverse primer in a total volume of 50 μL. For each StEP PCR condition (1 and 2), three reactions were prepared according to the following protocol: 98 °C for 3.5 min, 35 cycles of 98 °C for 30 s, 62 °C for 1 min, and 72 °C for 4 min, followed by 72 °C for 5 min and cooling to 4 °C. The purified products of the second PCR were assembled with the plasmid backbone in a total volume of 20 μL using the Gibson Assembly method.^[^ ^38^ ^]^ The procedure is described elsewhere.^[^ ^19^ ^]^ Two StEP libraries were mixed in equimolar amounts. After drop‐dialysis, electroporation of XL1‐Blue *E. coli* cells was performed.

For StEP library screening, 1 mL of autoinduction medium^[^ ^33^ ^]^ containing 100 μg mL^−1^ kanamycin was directly inoculated with single colonies of transformed BL21(DE3) in 96‐well deep well plates, covered with cell culture sheets, and incubated at 37 °C and 1000 rpm (Microtiterplate shaker TiMix 5, Edmund Bühler) for 5 h. The temperature was decreased to room temperature and protein expression was induced overnight. The activity assay was performed in a 384‐well plate format with a total reaction volume of 60 μL with diluted cell lysates (≈1380‐fold final dilution) containing overexpressed enzyme. 200 μL of cell lysates were transferred to 96‐well plates and incubated at 50 °C for 17 h (Heratherm oven, Thermo Scientific) under aluminum seal. Residual activity of the enzyme variants was measured at the final dilution of ≈690‐fold.

##### Enzyme Characterization

The half‐lives (*t*_1/2_), melting temperatures (*T*_m_), and isobutanol tolerance (*t*_1/2_ in isobutanol concentrations of 0 to 4.3% v/v) of the enzyme variants were determined as previously described.[^8^, ^39^] A temperature increment of +0.5 °C per 5 s from 4.0 to 100.0 °C was used for rapid initial screening and analysis of enzyme variants in *T*_m_, except that the incubation time was extended to 0.5 °C per 1 min for analysis of final variants FIN1 to FIN5 in lysates. The total turnover number (TTN) was estimated as follows^[^ ^40^ ^]^:

(1) $$\text{TTN}=\frac{k_{\text{cat, app}}}{k_{\text{d, app}}}$$

where $k_{\text{cat, app}}$ is the apparent turnover number [s^−1^] and $k_{\text{d, app}}$ is the apparent deactivation constant [s^−1^].

##### In Vitro Cascade for the Production of Ethanol from Glucose

The final enzyme concentrations were prepared as stated in Table S13, Supporting Information. The cascade reactions were performed in PCR tubes at 50 °C using an MJ Mini Thermal Cycler (Bio‐Rad). Each reaction mixture had a final volume of 200 μL and contained 5 mm NAD^+^, 0.5 mm TPP, 5 mm MgCl_2_, 100 mm d‐glucose, and 200 mm HEPES, pH 8.0. The reaction was started by adding the reaction mixture to the enzyme solution. At each timepoint, 20 μL of samples were taken and diluted 1:10 in 2.5 mm H_2_SO_4_ and filtered through 10 kDa centrifugal columns (VWR) before analysis. Substrate, intermediate, and product concentrations were quantified using the HPLC system and method as described previously.^[^ ^1^ ^]^

## Conflict of Interest

The authors declare no conflict of interest.

## Supporting information

Supplementary Material

## Data Availability

The data that support the findings of this study are available in the supplementary material of this article
