# Supplementary material for: Microfluidic‐Assisted Evolution of a Robust NAD+‐Dependent Enzyme with Improved Isobutanol Tolerance at Elevated Temperatures
Source: ChemSusChem. 2025 Jul 10;18(20):e202501120. doi: 10.1002/cssc.202501120 (PMC12548936; doi:10.1002/cssc.202501120)
Supplement: Supplementary file 1 — Supplementary Material [file CSSC-18-e202501120-s001.pdf]

Table of Contents

1. Experimental Information 1
2. Supplementary Tables 3
3. Supplementary Figures 14
4. Literature 23

1. Experimental Information

Purification Tag Engineering

Before starting with the generation of a new library (gene-wide DNA manipulation) and its screening, the re-assessment of the gene construct was considered to be important. In this regard, we specifically focused on the location and possible steric interactions of the hexahistidine tag. As shown previously, the location of a His_6 tag at the N- or C-terminus can significantly affect both the activity and stability of proteins, particularly *Hs*ALDH.[8c] The CHis wild-type form is 1.7-fold more active than its NHis counterpart. This significant catalytic advantage of the CHis form led to our initial decision to use *Hs*ALDH_Chis for engineering in the recent work. However, the T_m of the CHis form is only 50 °C, as opposed to 57 °C for the NHis form, which led to the compromise of using the variants at a lower cascade reaction temperature between 37 and 45 °C.[8c] Nevertheless, the *T*m increased to 54 °C after the introduction of the first mutation V284I to generate the template variant used in the present work. Since the stepwise thermostabilization of *Hs*ALDH maintained or even increased its high activity against 5 mM D-glyceraldehyde, we wondered if we could reassess the potential of the NHis counterpart without dramatically affecting the activity from our starting point of engineering. For this purpose, two modified N-terminal sequences were designed to test differences in flexibility, hydrodynamic volume, and length of the freely moving NHis tag. The first "GS-linker" replaced the thrombin cleavage site (Leu-Val-Pro-Arg-Gly-Ser) and the next His residue on the pET28a vector with a Gly-Ser linker (**Figure S13a**). The second "short" linker construct was removed from this sequence patch to generate a shorter NHis (**Figure S13a**).

The GS and short linkers showed similar characteristics in both specific activities and thermostability. They improved the specific activity of *Hs*ALDH_I36V_F147L_V284I_Nhis against 5 mM D-glyceraldehyde from 15 U mg^{-1} to up to 22 U mg^{-1}. Their $t_{1/2}$ values were estimated to be 12.7 hours (2^8-fold improvement over the wild type CHis), improving the value for the CHis variant of 8.1 hours almost to the level of the NHis variant with a $t_{1/2}$ of 14.3 hours (**Figure S13b**). Similarly, the T_m values for the NHis variant, GS and short linkers were approximately 63.5 °C, while that of the CHis variant was 61.0 °C (**Table S6**). Due to the advantages in both activity and stability compared to both CHis and NHis variants, we decided to use one of the new constructs, the GS linker, for further engineering.

Staggered Extension Process (StEP)

Four non-natural codon-optimized template genes were used on which selected mutations were artificially distributed (**Figure S14b; Table S8**): template 1 contains only the established mutations, V284I, I36V and F147L. Template 2 contains a total of 14 mutations, three established mutations and 11 candidate mutations. In addition, 11 selected candidate mutations were alternately distributed on template 3 and 4, *i.e.* Template 3 contains six mutations A39T, I137F, Y190F, A246T, K415M, and I434T besides the three established ones. In template 4, five additional intermediate mutations (F64Y, V158A, K221R, P371L,

and L432M) were integrated in addition to the three established mutations. In this way, five scenarios were enabled: (1) the integration of only a minimum number of mutations (template 1), (2) the generation of variants with higher mutation densities (template 2), (3) mutations in close proximity (template 2), (4) the generation of variants with moderate numbers of mutations (template 3 and template 4), (5) the separation of two mutations in close proximity (template 3 and template 4). The three established mutations are retained in any case of crossover events, and 11 new candidate mutations are present equally on the template genes, ensuring the same possibility of their introduction.

To further counteract biases in the introduction of mutations, we employed a second strategy by combining two different StEP PCRs (**Table S9**). By combining different crossover frequencies, a wide distribution of crossover numbers was obtained while still maintaining a large population of full-length mosaic amplicons and a higher DNA yield. Furthermore, the StEP primers annealed at sufficient distances outside the *Hs*ALDH gene to avoid lower crossover numbers at either end and higher crossover numbers in the middle of the fragment.

The identical DNA sequence patterns distributed on the template genes were edited by changing the codon triplets to avoid unspecific recombinations leading to deletions (**Figure S15**). The use of a highly accurate but slower polymerase, Accuzyme (Bioline GmbH), is a reason for longer annealing/extension times, but facilitated obtaining sufficient full-length progeny genes. These operations resulted in a significant reduction in the percentage of progeny genes with deletions from >75% to 22%. High PCR cycle numbers between 200 and 250 were chosen to counteract the longer annealing and extension times for increased accuracy at the expense of lower crossover frequency.

2. Supplementary Tables

Table S1. Activity landscapes of error-prone PCR (epPCR) libraries generated from *Hs*ALDH_V284I_Chis (labeled as wild-type (WT) as a reference point). epPCR libraries were generated with varying concentrations of $MnCl_2$ ranging from 0.06 to 1.00 mM. Each library was analyzed in a 96-well plate for activity landscapes and the variants were categorized into different classes: high (variants with >80% WT activity), intermediate (variants with 20≤80% WT activity) and very low (variants with <20% WT activity) activity. Clones with no growth were excluded from the calculation.

$MnCl_2$ (mM)	Variants with >80% WT activity (%)	Variants with 20≤80% WT activity (%)	Variants with <20% WT activity (%)
1.00	0.0	0.0	100.0
0.50	0.0	0.0	100.0
0.25	13.4	7.3	79.3
0.13	32.5	11.3	56.3
0.06	44.6	14.9	40.5

Table S2. Analysis of mutations introduced in the *Hs*ALDH_V284I_Chis epPCR library generated with 0.25 mM $MnCl_2$. Five clones were sequenced to estimate the average number of introduced mutations.

Number of amino acids	477
Number of exchanged nucleotides	5.4
Number of exchanged amino acids	4.2
Complexity of library	63,000
Transition/Transversion (unbiased: 0.5)	2.0
AT→GC/GC→AT (unbiased: 1.0)	1.3
AT→NN/GC→NN (unbiased: 1.0)	2.0

Table S3. AADS sorting data. Droplets were collected from 0 to 4.5 V.

**Voltage (V)**	**Number of Droplets**	**Percentage (%)**
0.05	0	0
0.15	0	0
0.25	0	0
0.35	0	0
0.45	0	0
0.55	0	0
0.65	0	0
0.75	0	0
0.85	0	0
0.95	0	0
1.05	0	0
1.15	0	0
1.25	0	0
1.35	0	0

1.45	0	0
1.55	0	0
1.65	0	0
1.75	0	0
1.85	0	0
1.95	0	0
2.05	0	0
2.15	0	0
2.25	0	0
2.35	0	0
2.45	0	0
2.55	1	7.68292E-07
2.65	0	0
2.75	0	0
2.85	0	0
2.95	1	7.68292E-07
3.05	1	7.68292E-07
3.15	1	7.68292E-07
3.25	0	0
3.35	0	0
3.45	0	0
3.55	0	0
3.65	2	1.53658E-06
3.75	1	7.68292E-07
3.85	5	3.84146E-06
3.95	33	2.53536E-05
4.05	79	6.06951E-05
4.15	136	0.000104488
4.25	242	0.000185927
4.35	273	0.000209744
4.45	297	0.000228183
4.55	455	0.000349573
4.65	622	0.000477878
4.75	482	0.000370317
4.85	507	0.000389524
4.95	767	0.00058928
5.05	1241	0.000953451
5.15	2150	0.001651828
5.25	3357	0.002579157
5.35	5811	0.004464546
5.45	11593	0.008906812
5.55	25245	0.019395538
5.65	50622	0.038892491
5.75	87936	0.067560549
5.85	124979	0.0960204

5.95	159797	0.1227708
6.05	181587	0.139511889
6.15	186916	0.143606118
6.25	170776	0.131205881
6.35	120569	0.092632231
6.45	58566	0.044995805
6.55	22816	0.017529356
6.65	11954	0.009184166
6.75	8543	0.006563521
6.85	4464	0.003429657
6.95	3350	0.002573779
7.05	3296	0.002532291
7.15	3302	0.002536901
7.25	3407	0.002617572
7.35	3291	0.00252845
7.45	3559	0.002734352
7.55	3913	0.003006328
7.65	4681	0.003596376
7.75	5707	0.004384644
7.85	7086	0.005444119
7.95	10301	0.007914179
8.05	5124	0.00393673
8.15	722	0.000554707
8.25	1	7.68292E-07
8.35	8	6.14634E-06
8.45	72	5.5317E-05
8.55	144	0.000110634
8.65	135	0.000103719
8.75	113	8.6817E-05
8.85	84	6.45366E-05
8.95	69	5.30122E-05
9.05	66	5.07073E-05
9.15	48	3.6878E-05
9.25	38	2.91951E-05
9.35	42	3.22683E-05
9.45	35	2.68902E-05
9.55	48	3.6878E-05
9.65	47	3.61097E-05
9.75	29	2.22805E-05
9.85	23	1.76707E-05
9.95	20	1.53658E-05
	1301588	1

Table S4. Occupancy of droplets by cells. Droplets were encapsulated according to the Poisson distribution with λ = 0.1.

Number of cells encapsulated in droplets	Poisson $P_{0.1}$	Droplets number	Droplets (%)	Droplets Sum (%)
Z=0	9.05E-01	1.18E+06	90.484%	90.484%
Z=1	9.05E-02	1.18E+05	9.048%	9.048%
Z=2	4.52E-03	5.89E+03	0.452%	0.4679%
Z=3	1.51E-04	1.96E+02	0.015%	
Z=4	3.77E-06	4.91E+00	0.000%	
Z=5	7.54E-08	9.81E-02	0.000%	

Table S5. Representative variants and their mutations from the second round of 96-well plate screening. Their selection was based on a comparison of their initial lysate activity and residual activity after 2 hours of incubation with the corresponding values of the template (containing the V284I mutation). Candidates of interest were expressed in small flasks containing 50 mL culture media and purified for characterization. Specific activities towards 5 mM (low substrate concentration) and 50 mM D-glyceraldehyde (high substrate concentration; activity at near saturation), residual activities after 0.5 hours of incubation at 50 °C and melting points (T_m) were measured. Furthermore, some selected single mutations were introduced into the template enzyme *Hs*ALDH_V284I_Chis by QuikChange mutagenesis and analyzed for their effects on specific activities, residual activities and T_m. The most significant increases in activity are highlighted in bold red. Those in thermostability are highlighted in the orange background (dark orange for particularly high thermostability). The significant decrease in thermostability is indicated by the blue background. Mutations identified more than once are marked in bold with the same color (green, light blue, etc.).

	Results from second round of screening		Variants expressed in flasks (from backup plates)				Analysis of single mutation (double mutants generated by QuikChange mutagenesis)				
Variant number	Activity (U mL^{-1})	Change in activity (%) 2h	Activity at 5 mM (%)	Activity at 50 mM (%)	Resid. activity 0.5 h (%)	ΔT_m (°C)	Introd. mutation	Activity at 5 mM (%)	Activity at 50 mM (%)	Resid. activity 0.5 h (%)	ΔT_m (°C)
WT	See right		122	126	1	-4.2	-	133	**138**	1	-4.2
**V284I (temp.)**	**29.3**	**0**	**100**	**100**	**3**	**0**	V284I	**100**	**100**	**3**	**0**
1	See right		**144**	126	45	+2.7	I33N	ND			
							I36V	**189**	134	83	+5.3
							F276Y	ND			
2	**41.2**	-10	**149**	139	1	-2.2	K53E	See left			
3	**39.1**	-17	94	97	1	-4.0	M90L	ND			
							L404P				
4	35.9	+8	**148**	**158**	3	-1.0	L432M*	See left			
5	35.7	-13	108	118	1	-3.5	L44M	ND			
							I355V				
6	35.3	-10	134	**149**	2	-1.8	P218L	See left			
7 (two clones)	34.9	+23	124	111	1	-2.8	Y306F*	156	113	4	-0.2
							**A414T***	**165**	113	1	-2.8

	Results from second round of screening		Variants expressed in flasks (from backup plates)				Analysis of single mutation (double mutants generated by QuikChange mutagenesis)				
Variant number	Activity (U mL^{-1})	Change in activity (%) 2h	Activity at 5 mM (%)	Activity at 50 mM (%)	Resid. activity 0.5 h (%)	ΔT_m (°C)	Introd. mutation	Activity at 5 mM (%)	Activity at 50 mM (%)	Resid. activity 0.5 h (%)	ΔT_m (°C)
8	34.8	+10	139	134	3	-0.2	**E317D**	108	85	3	-3.7
9	34.4	+12	140	127	3	-0.7	T393S	See left			
10 (three clones)	33.5	+11	**149**	**204**	29	+2.3	V158A	ND			
							P371L				
11	33.2	+22	131	122	23	+2.5	K415M*	See left			
12	33.0	+30	133	129	32	+2.8	**I137F***	**173**	121	47	+2.3
13 (two clones)	32.8	+15	139	129	5	-0.5	V304I	See left			
14 (two clones)	31.9	+18	123	116	27	+2.7	F64Y*	See left			
15 (three clones)	30.6	+16	ND				I284V	Reverted to WT			
							**A414T***	**165**	113	1	-2.8
16	30.2	+14	107	134	2	-0.7	S390P	See left			
**17**	30.1	+34	121	129	81	**+4.5**	K221R*	See left			
18	29.1	+34	ND				A272V	ND			
							**I434T***				
19	28.8	+24	ND				T247S	ND			
							**E317D**	108	85	3	-3.7
20	27.5	+20	ND				I36F	**174**	127	60	+2.8
							N297S	ND			
							L337H	ND			
21 (two clones)	26.0	+34	ND				A39T	ND			
22 (two clones)	25.0	+18	ND				**I137F***	**173**	121	47	+2.3
							Q292H	ND			
23	24.2	-19	47	28	1	-8.3	V206A	ND			
							H217Q				
							A237V				
24 (two clones)	24.0	+24	ND				T120I	ND			
							A184S				
							F296I				
25 (two clones)	23.2	+37	ND				A37T	ND			
							R46C				
							A246T				
26	22.3	+38	ND				L370M	ND			
							**I434T***				
27	20.8	-16	109	100	1	-4.5	S18L	ND			

	Results from second round of screening		Variants expressed in flasks (from backup plates)				Analysis of single mutation (double mutants generated by QuikChange mutagenesis)				
Variant number	Activity (U mL^{-1})	Change in activity (%) 2h	Activity at 5 mM (%)	Activity at 50 mM (%)	Resid. activity 0.5 h (%)	ΔT_m (°C)	Introd. mutation	Activity at 5 mM (%)	Activity at 50 mM (%)	Resid. activity 0.5 h (%)	ΔT_m (°C)
							T287S				
28	20.6	-17	ND				V206A	ND			
							P371S				
29	19.1	+19	ND				M70L	ND			
							M88T				
30	18.2	+33	ND				H38L	ND			
							Y190F				
							R354H				
							A424V				
31	13.3	+19	ND				D260Y	ND			
32	12.6	+25	ND				A17V	ND			
							**R330C***	114	94	10	+0.8
							N468I	ND			
33	11.9	-14	ND				P95T	ND			
							G383S				
							S429C				
34	1.8	+128	ND				K35E	ND			
							F147L*				
							A208T*				
							**R330C***	114	94	10	+0.8
							Q344R	ND			

Table S6. Characterization of selected QuikChange variants. *Hs*ALDH_Chis variants were generated by stepwise introduction of mutations into the wild-type enzyme (WT_Chis). The tag-engineered variants were also generated by shifting and modifying the terminal sequences. For characterization, specific activities at 5 mM D-glyceraldehyde concentration, melting points (T_m) and half-lives ($t_{1/2}$) upon incubation at 50 °C were determined. Specific activities were measured with 5 mM D-glyceraldehyde, 5 mM NAD^+, in 100 mM HEPES, pH 7.5, at 50 °C in triplicates (n=3). Standard deviation (SD) is shown. The color code shows the lowest activities in yellow. The middle range of activities, comparable to the WT enzyme, are shown in white and the highest activities are shown in green. T_m values were determined by Thermofluor analysis in 100 mM HEPES, pH 7.5, in triplicates (n=3). The $t_{1/2}$ was estimated by monitoring the activity decay at 50 °C (n=3) and fitting the data with an exponential decay equation. The highest stability (T_m and $t_{1/2}$) is shown in red, the middle in white and the lowest in blue. *Var. 12 and 17 are from **Table S5**.

	Mutations	Var. number	Activity against 5 mM D-glyceral. (U mg^{-1})	T_m (°C)	$t_{1/2}$ at 50 °C
Wild types	WT_Chis	-	23.7 ± 0.3	49.5 ± 0.5	3 minutes
	WT_Nhis	-	12.1 ± 1.5	57.2 ± 0.3	1.2 hours
Mutation 1	V284I_Chis	-	18.0 + 0.3	53.0 ± 0.0	~10 minutes
Mutation 2	K221R_V284I_Chis	17*	27.3 ± 0.4	57.5 ± 0.0	2.0 hours
	I36V_V284I_Chis	35	33.9 ± 0.4	58.3 ± 0.2	2.2 hours
	I36F_V284I_Chis	36	31.3 ± 3.4	55.8 ± 0.2	0.6 hours
	I137F_V284I_Chis	12*	31.1 ± 1.4	55.3 ± 0.2	0.4 hours
Mutation 3	I36V_K221R_V284I_Chis	37	32.3 ± 0.8	62.0 ± 0.0	3.5 hours
	I36V_V284I_I434T_Chis	38	32.6 ± 1.0	57.5 ± 0.0	3.8 hours
	I36V_I137F_V284I_Chis	39	34.5 ± 0.4	59.3 ± 0.2	3.1 hours
	I36V_V284I_A414T_Chis	40	35.5 ± 0.8	55.0 ± 0.0	2.9 hours
	I36V_V284I_Y306F_Chis	41	36.0 ± 0.9	58.0 ± 0.0	1.9 hours
	I36V_F64Y_V284I_Chis	42	32.6 ± 0.9	59.8 ± 0.2	2.9 hours
	I36V_V284I_K415M_Chis	43	33.9 ± 0.1	59.5 ± 0.0	2.7 hours
	I36V_V284I_L432M_Chis	44	28.9 ± 1.1	57.5 ± 0.0	2.2 hours
Mutations from "21D1"	I36V_F147L_V284I_Chis	45	26.4 ± 0.4	61.0 ± 0.0	8.1 hours
	I36V_A208T_V284I_Chis	46	32.1 ± 0.4	58.3 ± 0.2	2.0 hours
	I36V_V284I_Q344R_Chis	47	29.0 ± 2.1	58.5 ± 0.0	2.8 hours
Tag Engineering	I36V_F147L_V284I_Nhis	48	14.5 ± 0.5	63.5 ± 0.0	14.3 hours
	I36V_F147L_V284I_GS-linker_Nhis	49	22.0 ± 0.8	63.7 ± 0.3	12.7 hours
	I36V_F147L_V284I_short_Nhis	50	21.4 ± 0.7	63.5 ± 0.0	12.7 hours

Table S7. Substrate spectrum of the *Hs*ALDH variants. The variants were analyzed for possible shifts in substrate selectivity by measuring specific activities towards 5 mM concentrations of D-glyceraldehyde and other aldehyde species, as well as towards 50 mM D-glyceraldehyde. Reaction mixtures also contained 5 mM NAD^+ in 100 mM HEPES, pH 7.5, and measurements were performed in triplicates (n=3). Activities are given as a percentage of the wild-type (*Hs*ALDH_Chis) activity towards 5 mM D-glyceraldehyde (shown in white at the top left). The highest activities are shown in dark green, the lowest in yellow. The unspecific activity of the mutant variants towards isobutyraldehyde remained below 3%.

		5 mM D-glyceraldehyde	50 mM D-glyceraldehyde	5 mM isobutyraldehyde	5 mM acetaldehyde	5 mM formaldehyde	5 mM glycolaldehyde
	WT	100	255	1	93	27	151
Template	V284I	82	193	1	70	20	118
Var. 17	K221R_V284I	115	293	3	86	20	169
Var. 35	I36V_V284I	130	296	2	83	26	147
Var. 37	I36V_K221R_V284I	136	226	2	88	22	123
Var. 38	I36V_V284I_I434T	138	200	2	83	27	165
Var. 39	I36V_I137F_V284I	146	214	2	88	26	153
Var. 40	I36V_V284I_A414T	150	214	2	80	27	157
Var. 41	I36V_V284I_Y306F	152	235	2	83	26	150
Var. 42	I36V_F64Y_V284I	138	218	2	83	26	148
Var. 43	I36V_V284I_K415M	143	221	2	84	27	155
Var. 44	I36V_V284I_L432M	122	187	1	65	20	118
Var. 45	I36V_F147L_V284I	109	194	1	69	27	150
Var. 46	I36V_A208T_V284I	136	248	2	63	32	164
Var. 47	I36V_V284I_Q344R	122	228	2	86	27	150

Activity compared with WT for D-GA (%): 300, 250, 200, 150, 100, 50

Table S8. Characterization of the template variants used for the Staggered Extension Process (StEP). Templates 1 to 4 (codon-optimized genes) were generated as illustrated in **Figure S14**. The number of amino acid exchanges contained in each template enzyme, specific activities against 5 mM D-glyceraldehyde, half-lives at 50 °C ($t_{1/2}$), and protein solubility are also shown. Template 1 (codon optimized) and *Hs*ALDH_I36V_F147L_V284I_Nhis (nucleotide sequence from the original host) encode the same amino acid sequences, but show differences in activity and stability (possibly a folding issue). Specific activities were measured using 5 mM D-glyceraldehyde and 5 mM NAD^+ in 100 mM HEPES, pH 7.5, at 50 °C in triplicates (n=3), and the activity decrease at 50 °C was followed in triplicates (n=3) under the same assay conditions. Protein solubility was checked by SDS-PAGE after cell lysis.

Plasmid name	Number of mutations	Codon-optimization	Specific activity (U mg^{-1})	$t_{1/2}$ at 50 °C (h)	Protein solubility
Template 1	3	Yes	11.8 ± 1.0	10.3	Good
Template 2	14	Yes	2.9 ± 0.3	6.2	Very poorly soluble
Template 3	9	Yes	9.6 ± 0.2	8.6	Slightly soluble
Template 4	8	Yes	20.9 ± 1.7	5.9	Partially soluble
*Hs*ALDH_I36V_F147L_V284I_Nhis	3	No	14.5 ± 0.5	14.3	Good

Table S9. PCR programs for StEP. DNA templates 1 to 4 were mixed in equimolar ratio and subjected to StEP PCRs 1 and 2.

StEP1		min:sec	
1.	95 °C	04:00	
2.	94 °C	00:20	
3.	55 °C	00:30	
4.	72 °C	00:20	
5.	Go to 2.		x 200
6.	4 °C	∞	

StEP 2		min:sec	
1.	95 °C	04:00	
2.	94 °C	00:20	
3.	55 °C	00:20	
4.	Go to 2.		x 250
5.	4 °C	∞	

Table S10. Lysate activity. The StEP library and template 1 as reference were used to transform *E. coli* by electroporation, and the cells were cultured in different media (TB medium with IPTG for induction of gene expression or auto-induction medium) for protein expression. Before harvesting the cells, the cell density was measured in optical density (OD_{600}) at a wavelength of 600 nm. The activities of the different expression conditions were compared by normalization to culture volume and OD_{600}.

Expression	Medium/Induction	End OD_{600}	Activity (U mL^{-1} $OD_{600}{}^{-1}$)
Template 1	TB/IPTG	20.4	1.25 ± 0.03
Template 1	Autoinduction	27.8	0.94 ± 0.14
StEP library	TB/IPTG	12.8	0.32 ± 0.04
StEP library	Autoinduction	18.7	0.46 ± 0.03

Table S11. Screening of the StEP library. Initial lysate activity (0 h (%)) and residual activity after 17 hours of incubation at 50 °C (17 h (%)) of 11 unique variants of *Hs*ALDH_I36V_F147L_V284I_Nhis (T1 = template 1) identified from the 96-well plate screening are shown and compared with the T1 activities. The variants were expressed in 50 mL autoinduction medium and activity measurements were performed on lysates (Activity (U mL^{-1})). Activities (U mL^{-1} OD^{-1}) normalized to the final cell density (End OD) are also shown. The color codes show higher activities in green and lower activities in orange-yellow, higher stability in red and lower stability in blue. Introduced mutations were identified by sequencing and are colored in yellow. The number of amino acid exchanges at each position was counted. Some additional (silent or missense) mutations were also identified. Three positions 158, 221 and 432, marked in red, were selected for further investigation.

							Position of exchange											Additional exchanges
Variant	0 h (%)	17 h (%)	$t_{1/2}$ (h)	Activity (U mL^{-1})	Activity (U mL^{-1} OD^{-1})	End OD	39	64	137	**158**	190	**221**	246	371	415	**432**	434	
T1	100	100	9.8	4.8	0.21	22.6	A	F	I	V	Y	K	A	P	K	L	I	
T2							T	Y	F	A	F	R	T	L	M	M	T	
**51**	78	430	13.0	4.1	0.41	10.1	A	F	I	V	Y	R	A	P	K	L	I	
**52**	194	175	8.4	7.4	0.29	25.7	A	Y	I	V	Y	K	A	P	K	L	I	
**53**	92	187	9.7	2.7	0.26	10.7	A	F	I	V	Y	K	A	P	K	L	I	G11D
**54**	37	163	7.5	2.3	0.11	21.4	T	F	I	V	Y	K	A	P	K	L	I	
**55**	72	119	9.4	2.1	0.22	9.4	T	Y	I	A	Y	K	A	P	K	L	I	
**56**	142	115	8.4	5.0	0.23	21.6	A	F	I	V	Y	R	T	P	K	L	I	
**57**	67	187	8.5	4.8	0.19	24.9	A	F	F	V	F	R	A	P	K	L	I	
**58**	75	160	10.8	2.8	0.27	10.1	A	Y	I	A	Y	K	A	P	K	L	I	
**59**	57	142	10.0	1.7	0.19	9.0	T	F	F	V	F	K	A	P	K	M	I	
						Sum:	**3**	**3**	**2**	**2**	**2**	**3**	**1**	**0**	**0**	**1**	**0**	

Table S12. Computational analysis of mutation effects. Changes in Gibbs free energy ΔΔG were predicted by the tools DDMut,[3d] Dynamut,[15] and Fireprot v2.0.[16] DDMut was performed using a modeled monomer and multimer (in parentheses) structures. Fireprot v2.0 was performed using the model structure or sequence (in parentheses). Protein structures were modeled by AlphaFold 3.[14]

Mutations	DDMut ΔΔG (kcal/mol) Monomer (Multimer)	Dynamut ΔΔG (kcal/mol)	Fireprot v2.0 — Fold X $\Delta G_{WT}-\Delta G_{var}$ (kcal/mol) Structure (Sequence)
I36V	−0.41 (−0.75)	−1.10	0.25 (0.51)
F147L	−0.56 (−1.06)	−0.97	ND
V158A	−2.56 (−2.53)	−0.14	ND
K221R	−0.36 (−0.24)	**+0.08**	0.75 (0.45)
V284I	−0.19 (−0.11)	**+0.57**	ND
L432M	−0.24 (−0.32)	**+0.54**	−0.09 (−0.12)
I36V_F147L_V284I	−0.90 (ND)	ND	ND

Table S13. Final concentration of enzymes used in the glucose to ethanol cascade reactions.

Enzyme	Enzyme annotation	Organism	NCBI protein accession number	Final concentration (mg mL^{-1})	Reference
*Bs*GDH	Glucose dehydrogenase	*Bacillus subtilis*	WP_003246720.1	0.016	[1]
*Pu*DHT	Dihydroxy-acid dehydratase	*Paralcaligenes ureilyticus*	WP_132585145.1	5.950	
*Pt*KDGA	2-keto-3-deoxygluconate aldolase	*Picrophilus torridus*	WP_048059513.1	0.620	[2]
*Hs*ALDH variants	NAD^+-dependent succinate-semialdehyde dehydrogenase	*Herbaspirillum seropedicae Z67*	WP_013233459.1	8.000	This study [8c]
*Ap*PDC	Pyruvate decarboxylase	*Acetobacter pasteurianus*	WP_141376382.1	0.250	[13d]
BstADH	Alcohol dehydrogenase	*Bacillus stearothermophilus*	KFL15473.1	2.740	[3]

3. Supplementary Figures

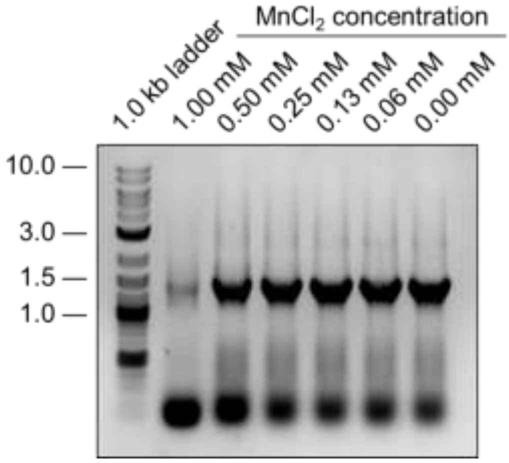

Figure S1. epPCR using the *Hs*ALDH_V284I_Chis plasmid as a template. $MnCl_2$ concentrations were varied from 0 to 1.00 mM to adjust the mutation efficiency.

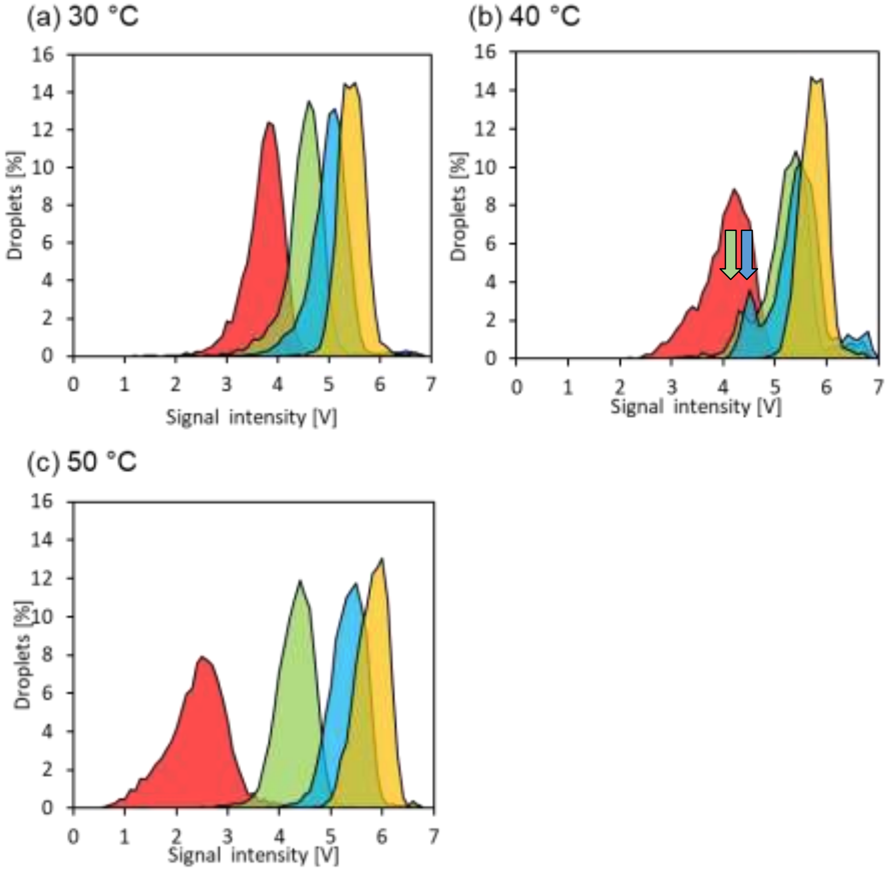

Figure S2. Testing of the incubation temperature and the time for droplet screening. Droplets containing *Hs*ALDH_V284I_Chis (template) and reaction mixtures were incubated at 30 (a), 40 (b), and 50 °C (c), and absorbance signals were monitored at different time points; directly after the encapsulation (orange), after 1.5 hours (blue), four hours (green), and 23 hours (red). Incubation of the droplets at 40 °C (b) resulted in optimal separation of enzyme-containing droplets (small left peak; indicated by arrows) from empty droplets (right major peak).

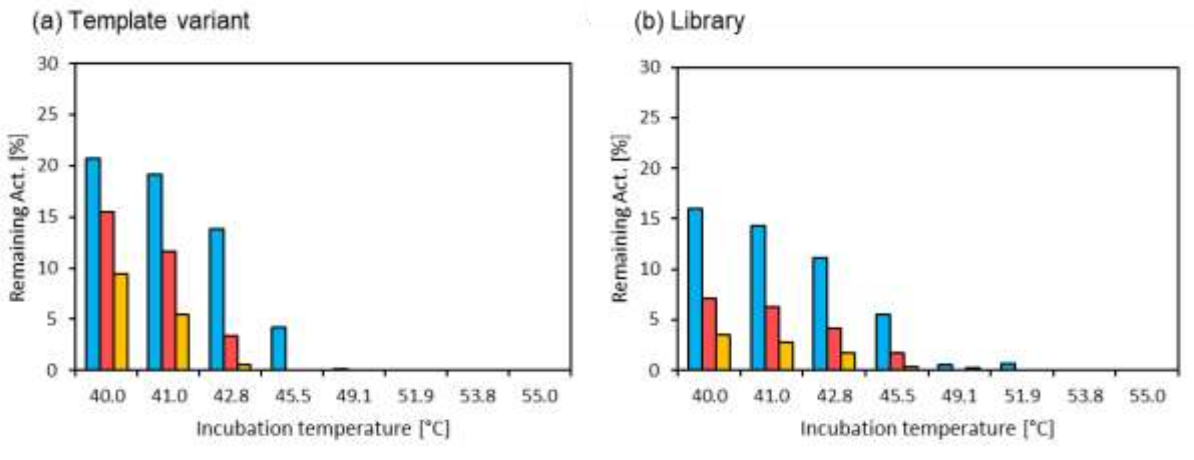

Figure S3. Temperature-dependent inactivation of the template variant *Hs*ALDH_V284I_Chis (a) and the epPCR library (b). Residual activities were measured after incubation in the thermocycler for one (blue), four (red) and six hours (orange) at different temperatures between 40.0 and 55.0 °C.

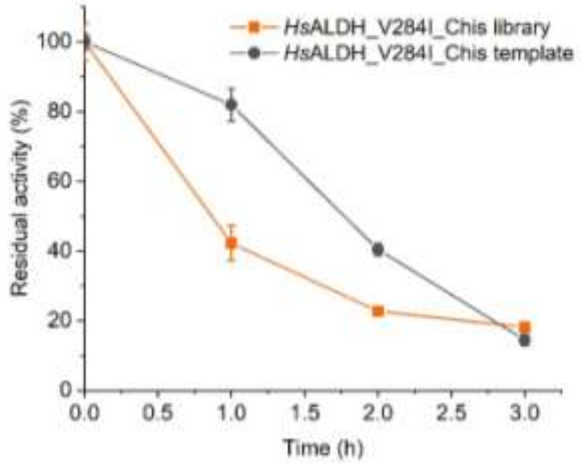

Figure S4. Comparison of the stability at 45 °C between *Hs*ALDH_V284I_Chis and the *Hs*ALDH_V284I_Chis library. Compared to the library, the activity of the template enzyme *Hs*ALDH_V284I_Chis (gray) decreased faster after one hour of incubation. The measurements of residual activities were performed with 5 mM D-glyceraldehyde, 5 mM NAD^+, 100 mM HEPES, pH 7.5 in triplicates (n=3) and the error bars represent the SD.

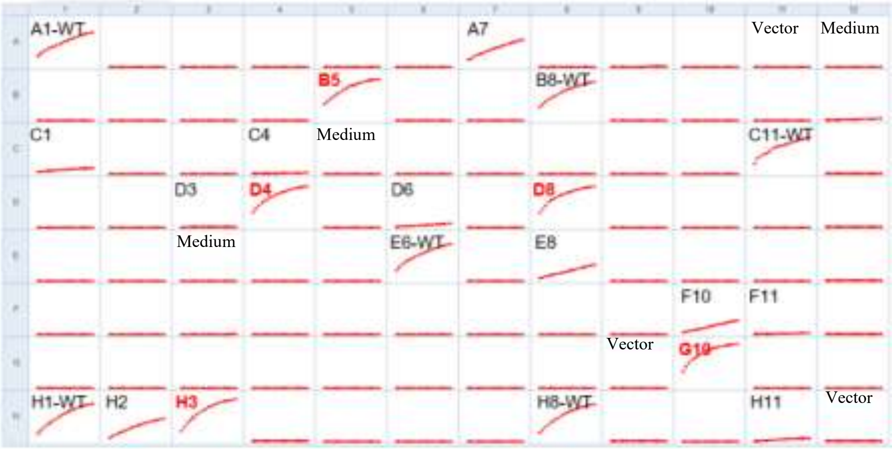

Figure S5. Analysis of the recovered hits on a 96-well plate. Activity measurements are shown.

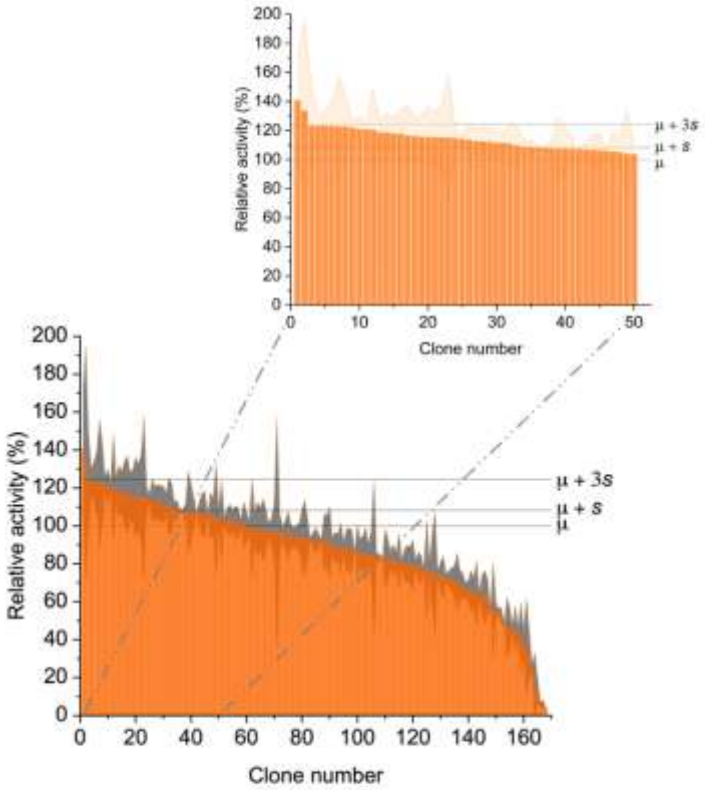

Figure S6. Activity landscape of the 168 selected clones from the *Hs*ALDH_V284I_Chis library in the second round of rescreening. The mean activity of the template is marked with a μ of 100%. The threshold for hits with μ + 3*s* is 125%. The top 50 clones are shown on the upper right. Activities were measured in lysates with 5 mM D-glyceraldehyde, 5 mM NAD^+, 100 mM HEPES, pH 7.5 in triplicate (n=3).

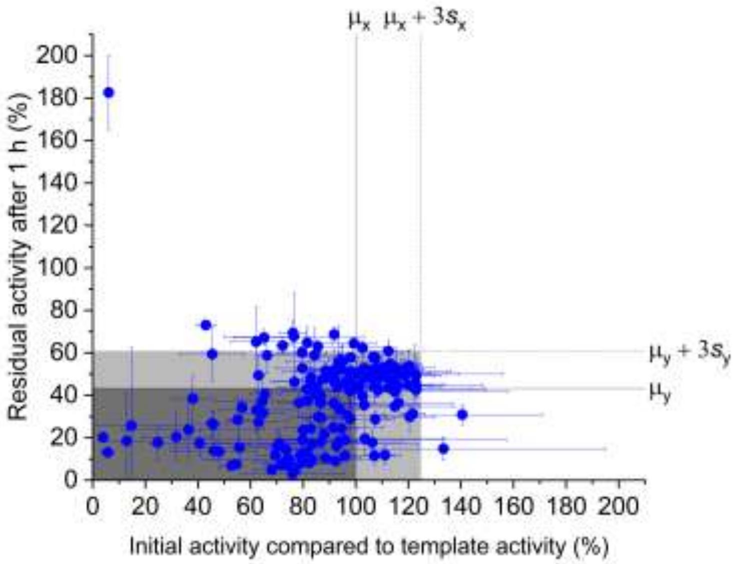

Figure S7. Second re-screening of the epPCR library (with residual activities after one hour). Initial activities as a percentage of the activity of the template are plotted against residual activities measured after one hour of incubation at 50 °C. The mean activity of the template and the threshold for hits are shown as μ_x = 100% and $\mu_x + 3s_x$ = 125% for the first measurements at t = 0 h, μ_y = 43% and $\mu_y + 3s_y$ = 61% for the second measurements at t = 1 h. Activities were measured in lysates using 5 mM D-glyceraldehyde, 5 mM NAD^+, 100 mM HEPES, pH 7.5

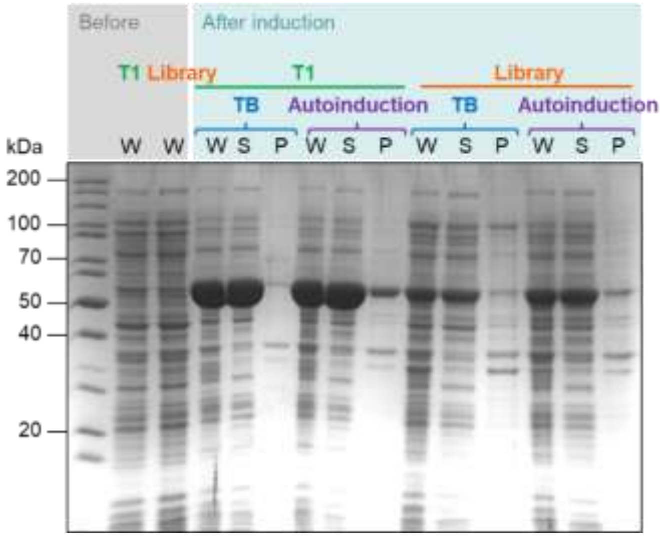

Figure S8. SDS-PAGE to analyze the expression and protein solubility of the StEP library. The StEP library (library) and template 1 (*Hs*ALDH_I36V_F147L_V284I_Nhis; marked as T1) for comparison were expressed in TB medium with IPTG induction or in autoinduction medium. Whole cell lysates (W), soluble protein fractions (S) and insoluble protein pellets (P) obtained after induction of gene expression are shown in comparison with the whole cell lysates before induction.

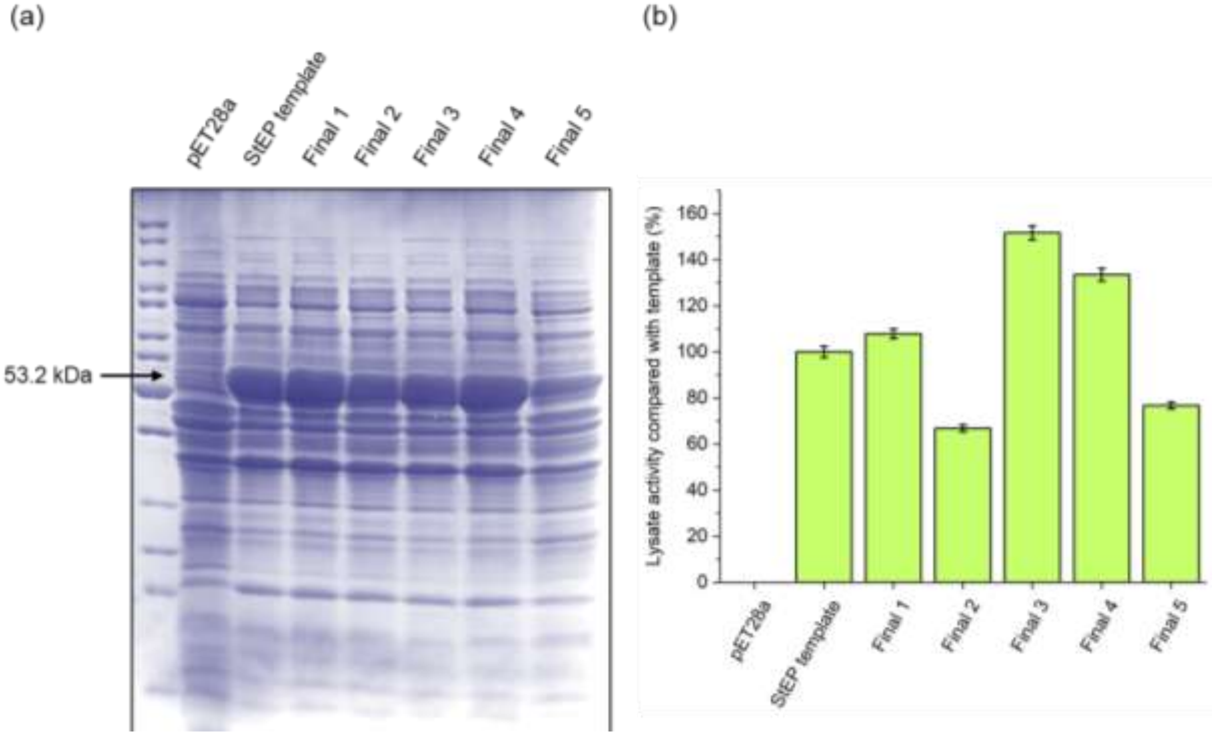

Figure S9. Expression (a) and activity (b) of the variants generated based on the StEP screening. (a) Expression of the *Hs*ALDH variants was examined by SDS-PAGE and compared with the template enzyme. The empty vector pET28a was used as a negative control. (b) The activity of the HsALDH variants was measured in lysates (n=3) using 5 mM D-glyceraldehyde and 5 mM NAD^+ in 100 mM HEPES, pH 7.5 at 50 °C and compared with the activity of the template. Error bars indicate SD.

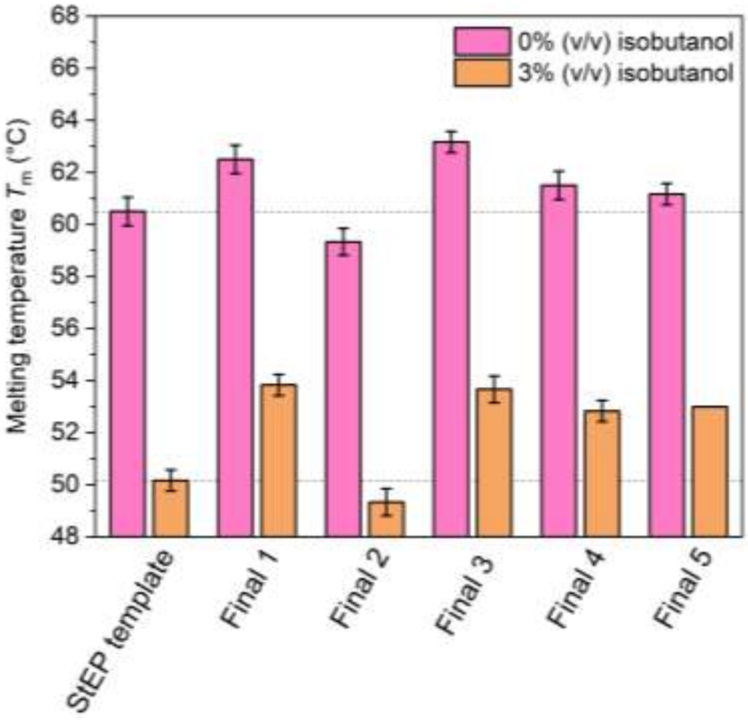

Figure S10. Melting temperature T_m of *Hs*ALDH variants with a GS linker measured in lysates. T_m was measured in the absence or presence of 3% (v/v) isobutanol by increasing the temperature by 0.5 °C per one minute from 4.0 to 100.0 °C.

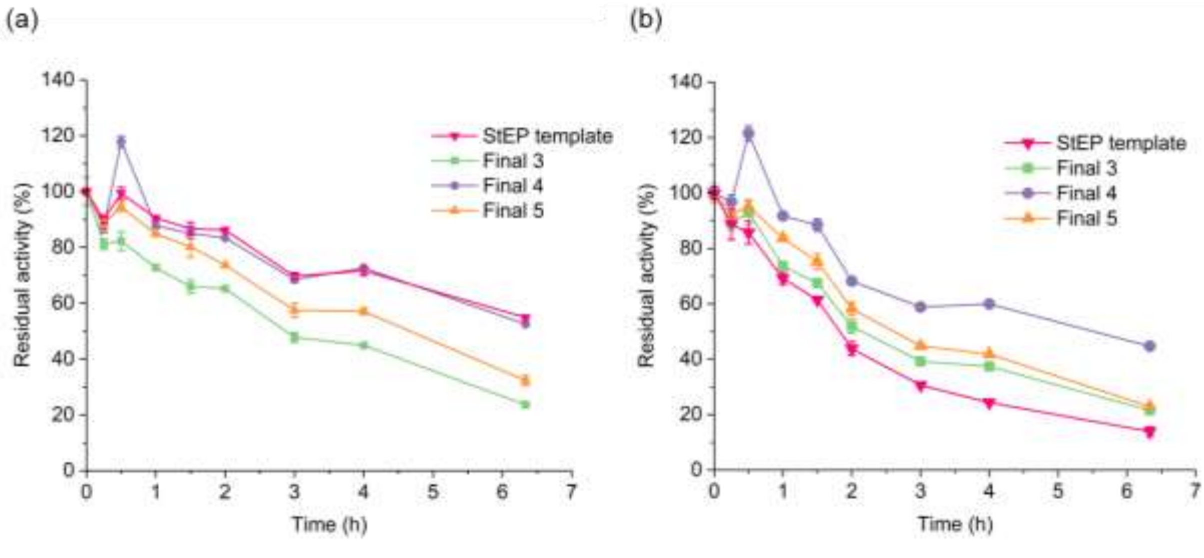

Figure S11. Activity decay of StEP variants in 2% (v/v) (a) and 3% (v/v) isobutanol (b) at 50 °C. Activities were monitored using 5 mM D-glyceraldehyde and 5 mM NAD^+ in 100 mM HEPES, pH 7.5 in triplicate (n=3). Error bars indicate SD.

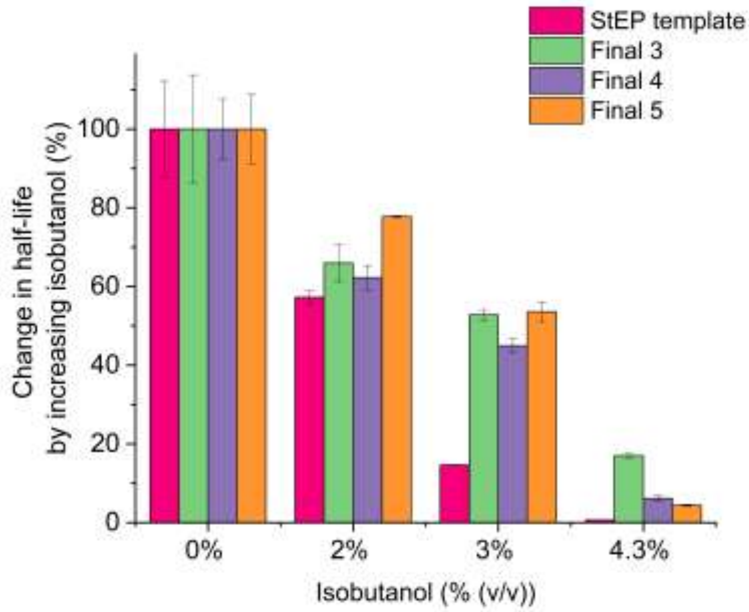

Figure S12. Influence of increasing isobutanol concentration on the half-life $t_{1/2}$. The StEP template, *Hs*ALDH_36V_F147L_V184I_GS-linker_Nhis, and var. 3–5 were compared in the susceptibility of their stability ($t_{1/2}$) to increasing isobutanol concentrations up to 4.3%.

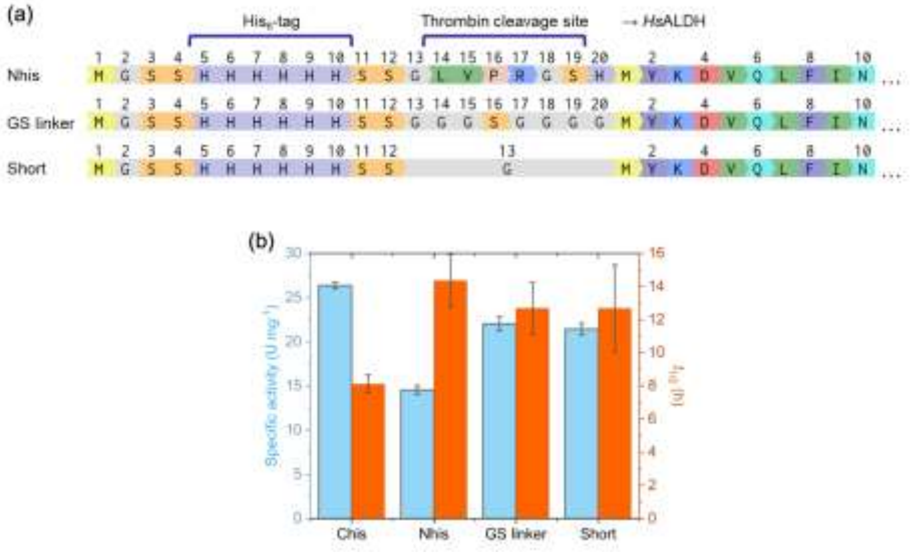

Figure S13. Optimization of the N-terminal hexahistidine tag for activity and stability. (a) Engineered N-terminal sequences (GS linker, Short) are shown in comparison to the original sequence (NHis). For *Hs*ALDH_I36V_F147L_V284I_NHis (not codon optimized), the thrombin cleavage site at the NHis was replaced by a glycine-serine linker (GS linker) or removed (Short). (b) Specific activities measured with 5 mM d-glyceraldehyde (light blue) and half-lives at 50 °C (orange) of four *Hs*ALDH_I36V_F147L_V284I variants with different hexahistidine tag sequences are compared. Specific activities were measured with 5 mM d-glyceraldehyde and 5 mM NAD^+ in 100 mM HEPES, pH 7.5, at 50 °C in triplicates (n=3) and error bars represent SD. Half-lives were estimated by monitoring the decrease in activity at 50 °C (n=3) using an exponential decay equation for fitting.

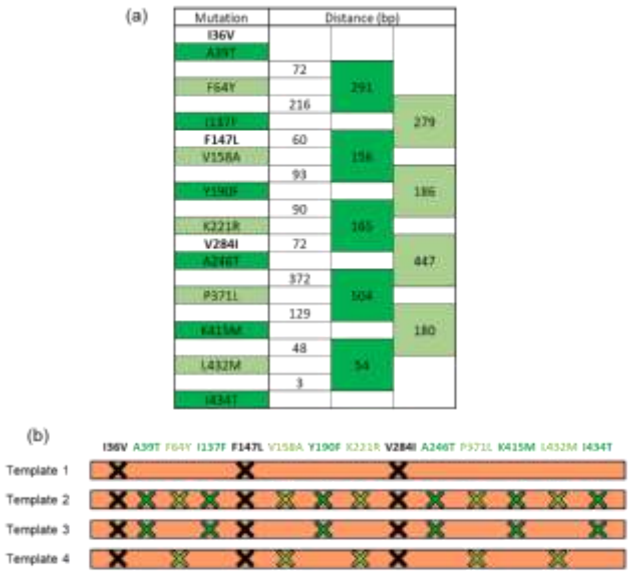

Figure S14. Schematic representation of the StEP for *in vitro* mutational recombination. (a) From the second round of re-screening, 11 mutations were selected for possible introduction into codon-optimized *Hs*ALDH_I36V_F147L_V284I_Nhis by StEP recombination. Each distance between the selected mutations is shown in base pairs (bp). (b) The mutations were distributed on template plasmids 1 to 4. Template 1 contains only the established mutations I36V, F147L and V284I. Template 2 contains all 11 mutations in addition to these. Furthermore, the 11 mutations were distributed alternately on Templates 3 and 4.

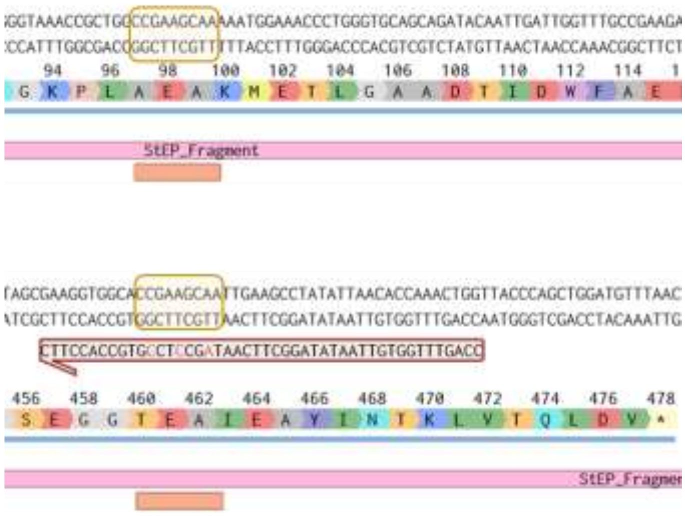

Figure S15. One of three pairs identified as identical DNA sequence patterns (indicated by yellow surroundings). One site of the pair (lower sequence) was edited to prevent deletions during the StEP PCR (the editing primer is shown in red).

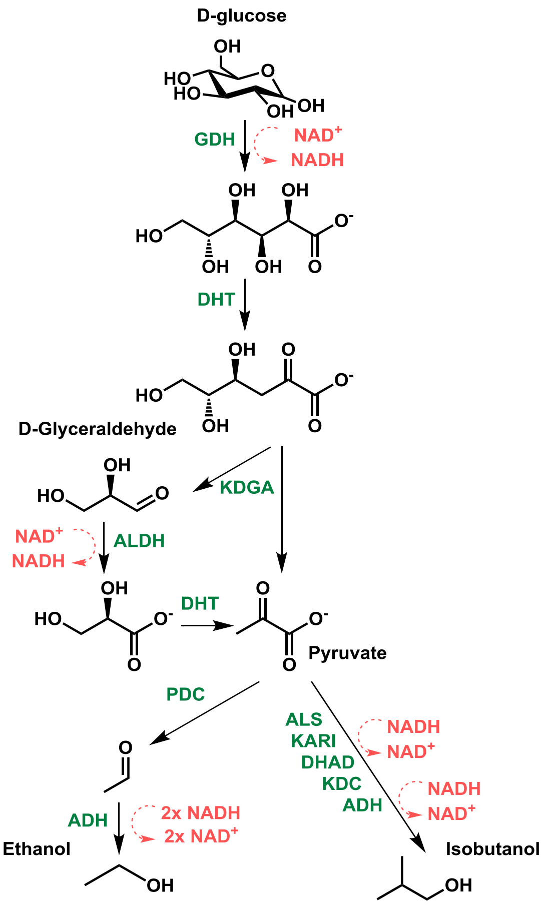

Figure S16. Scheme of the minimal cell-free enzyme cascades for the production of ethanol (left) and isobutanol (right).

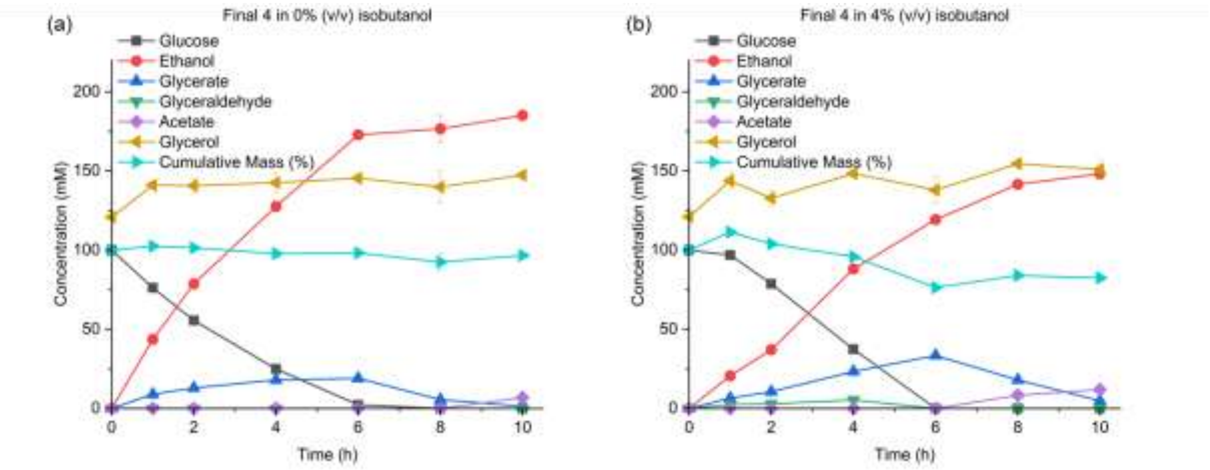

Figure S17. Time course of substrate, intermediate and product concentrations in the D-glucose to ethanol cascade reaction with final 4 variant in 0% (v/v) (a) and 4% (v/v) (b) isobutanol.

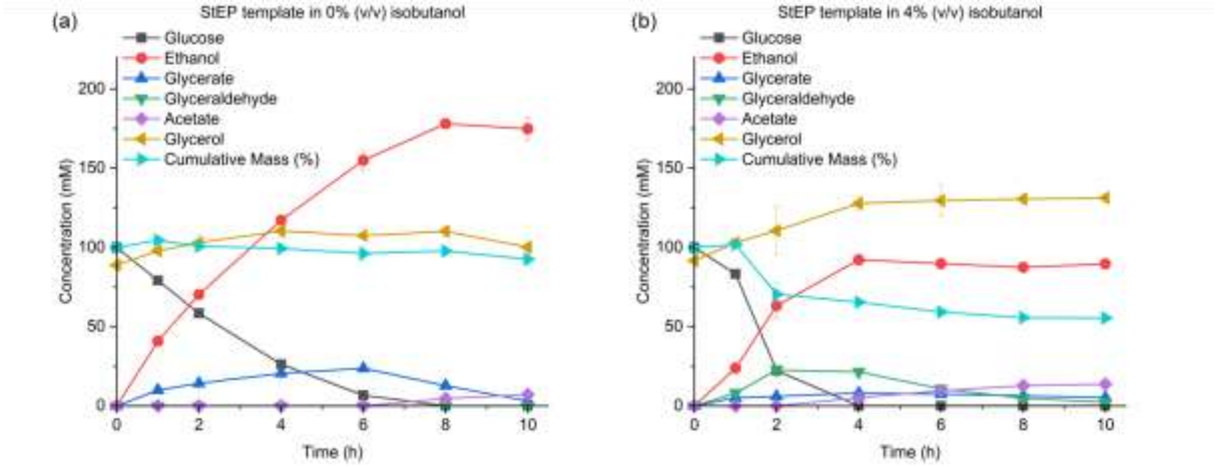

Figure S18. Time course of substrate, intermediate and product concentrations in the D-glucose to ethanol cascade reaction with StEP template variant in 0% (v/v) (a) and 4% (v/v) (b) isobutanol.

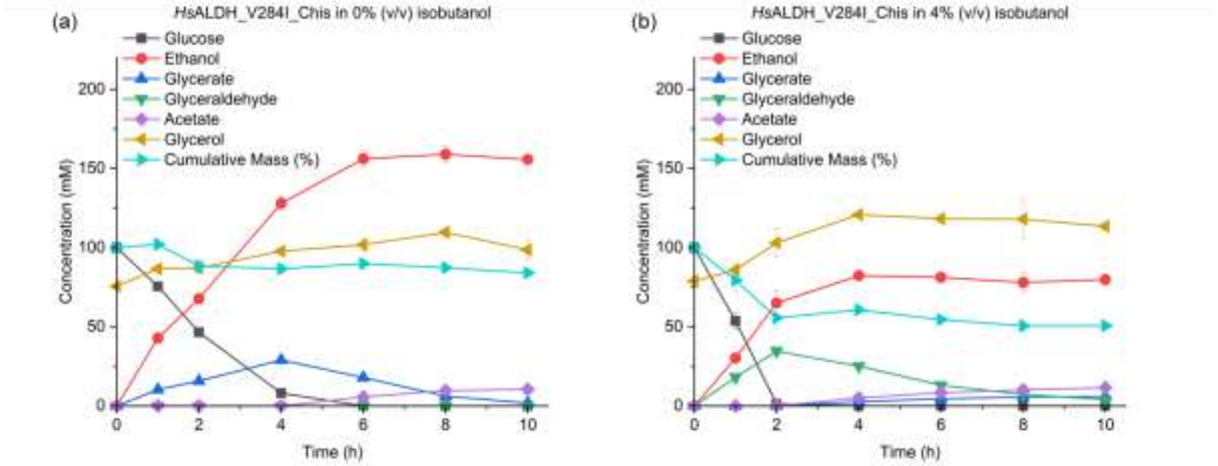

Figure S19. Time course of substrate, intermediate and product concentrations in the D-glucose to ethanol cascade reaction with *Hs*ALDH_V284I_Chis variant in 0% (v/v) (a) and 4% (v/v) (b) isobutanol.

4. Literature

[1] E. Vazquez-Figueroa, J. Chaparro-Riggers, A. S. Bommarius, *Chembiochem* **2007**, *8*, 2295-2301.

[2] M. Reher, T. Fuhrer, M. Bott, P. Schönheit, *J. Bacteriol.* **2010**, *192*, 964-974.

[3] A. Guagliardi, M. Martino, I. Iaccarino, M. D. Rosa, M. Rossi, S. Bartolucci, *Int. J. Biochem. Cell Biol.* **1996**, *28*, 239-246.
